# A whole-blood RNA transcript-based gene signature is associated with the development of CTLA-4 blockade-related diarrhea in patients with advanced melanoma treated with the checkpoint inhibitor tremelimumab

**DOI:** 10.1186/s40425-018-0408-9

**Published:** 2018-09-18

**Authors:** Philip Friedlander, Kevin Wood, Karl Wassmann, Alan M. Christenfeld, Nina Bhardwaj, William K. Oh

**Affiliations:** 1 Division of Hematology and Medical Oncology, Tisch Cancer Institute, Icahn School of Medicine at Mount Sinai Hospital, New York, NY USA; 2 Division of Hematology and Medical Oncology, Valley Hospital, Ridgewood, NJ USA; 3 CPS Companion Diagnostics, Cambridge, MA USA; 4 Parker Institute of Cancer Immunotherapy, San Francisco, CA USA

**Keywords:** Melanoma, Immunotherapy, irAE, Biomarker, CTLA-4, Diarrhea, Tremelimumab, Predictive

## Abstract

**Background:**

Anti-CTLA-4 immune checkpoint blockade is associated with immune-related adverse events (irAEs). Grade 3–4 diarrhea/colitis is the most frequent irAE requiring treatment discontinuation. Predicting high-risk diarrhea/colitis patients may facilitate early intervention, limit irAE severity, and extend treatment duration. No biomarkers currently predict for anti-CTLA-4 immunotherapy related severe diarrhea.

**Methods:**

Whole-blood was collected pre-treatment and 30 days post-treatment initiation from patients with stage III or IV unresectable melanoma who received 15 mg/kg tremelimumab at 90 day intervals in two clinical trials. The discovery dataset was a phase II study that enrolled 150 patients between December 2005 and November 2006. The validation dataset was a phase III study that enrolled 210 patients between March 2006 and July 2007. RT-PCR was performed for 169 genes associated with inflammation, immunity, CTLA-4 pathway and melanoma. Gene expression was correlated with grade 0–1 versus grade 2–4 diarrhea/colitis development.

**Results:**

Pre-treatment blood obtained from the discovery dataset (*N* = 150) revealed no gene predictive of diarrhea/colitis development (*p* < 0.05). A 16-gene signature (CARD12, CCL3, CCR3, CXCL1, F5, FAM210B, GADD45A, IL18bp, IL2RA, IL5, IL8, MMP9, PTGS2, SOCS3, TLR9 and UBE2C) was identified from 30 days post-tremelimumab initiation blood that discriminated patients developing grade 0–1 from grade 2–4 diarrhea/colitis. The 16-gene signature demonstrated an AUC of 0.814 (95% CI 0.743 to 0.873, *p* < 0.0001), sensitivity 42.9%, specificity 99.2%, positive predictive value (PPV) 90.0%, and negative predictive value (NPV) 91.4%. In the validation dataset (*N* = 210), the 16-gene signature discriminated patients developing grade 0–1 from grade 2–4 diarrhea/colitis with an AUC 0.785 (95% CI 0.723 to 0.838, *p* < 0.0001), sensitivity 57.1%, specificity 84.4%, PPV 57.1% and NPV 84.4%.

**Conclusion:**

This study identifies a whole-blood mRNA signature predictive of a clinically relevant irAE in patients treated with immune checkpoint blockade. We hypothesize that immune system modulation induced by immune checkpoint blockade results in peripheral blood gene expression changes that are detectable prior to clinical onset of severe diarrhea. Assessment of peripheral blood gene expression changes in patients receiving anti-PD-1/PD-L1 immunotherapy, or combination anti-CTLA4 and anti-PD-1/PD-L1 immunotherapy, is warranted to provide early on-treatment mechanistic insights and identify clinically relevant predictive biomarkers.

**Trial registration:**

Clinicaltrials.gov, NCT00257205, registered 22 November 2005

**Electronic supplementary material:**

The online version of this article (10.1186/s40425-018-0408-9) contains supplementary material, which is available to authorized users.

## Background

In the past few years, immune checkpoint blockade has changed the treatment landscape for many advanced malignancies. Currently, antibodies directed against three immune checkpoints, PD-1, PD-L1, and CTLA-4, have received regulatory approval in various parts of the world and have been integrated into standard practice guidelines. Enthusiasm for immune checkpoint blockade has been based largely on the ability of these therapies to induce durable benefit in a subset of patients. However, a risk of treatment with immune checkpoint blockade is the development of serious immune-related adverse events (irAEs) which can affect multiple organ systems. Therefore, attempts have been made to develop predictive biomarkers. To date, such efforts have focused predominantly on biomarkers predictive of treatment response while biomarkers associated with treatment-related toxicity have been underexplored.

CTLA-4 is a protein expressed on the surface of activated T-cells and, when bound to B7 on antigen-presenting cells, prevents T-cell co-stimulation [1]. Treatment of unresectable or stage IV melanoma patients who received prior chemotherapy with the investigational CTLA-4 inhibitor tremelimumab as part of a phase II study demonstrated a 6.6% objective response rate. Treatment related adverse events developed in 77% of patients with 21% of patients developing greater than grade 2 adverse events. Diarrhea of any grade developed in 40% of patients with 11% developing grade 3 or greater diarrhea. A randomized phase III study comparing treatment with tremelimumab versus chemotherapy in treatment naïve unresectable or stage IV melanoma patients failed to demonstrate survival benefit with tremelimumab although 10.7% of patients developed a treatment response [2]. Tremelimumab associated diarrhea or colitis developed in 51% of patients with 18% developing grade 3 or greater diarrhea.

Treatment of patients with unresectable or stage IV melanoma with the CTLA-4 inhibitor ipilimumab conferred overall survival benefit leading to Food and Drug Administration (FDA) approval in 2011. In the registration trial, 80% of patients developed ipilimumab-related all-grade toxicity, with 19.1% and 3.8% of patients developing grade 3 or 4 toxicities, respectively [1]. Diarrhea developed in 27.5% of ipilimumab monotherapy treated patients, with 7.6% and 0% developing grade 3 or 4 diarrhea, respectively. Colitis developed in 7.6% of patients, with 5.3% of patients developing grade 3 colitis. Diarrhea and/or colitis is the most common irAE related to CTLA-4 blockade that requires medical intervention with high dose corticosteroids and at times hospitalization and, in steroid refractory cases, anti-TNF-α-therapy. Ipilimumab is administered every 21 days, and on average patients receive 3 doses before developing diarrhea and other gastrointestinal irAEs, but the time of onset is variable and the severity ranges from moderate (grade 2) to severe (grade 3) and life-threatening (grade 4) [3].

Subsequently, two inhibitors of PD-1, nivolumab and pembrolizumab, were FDA approved for treatment of unresectable or stage IV melanoma [4, 5]. PD-1 expressed on the surface of tumor infiltrating T-cells binds to PD-L1 expressed on tumor cells leading to functional inhibition of the T-cells. Both of the PD-1 inhibitors confer 35–40% response rates with grade 3 or higher immune mediated toxicity developing in 16–20% of patients. Combining CTLA-4 and PD-1 blockade through concomitant treatment with ipilimumab and nivolumab leads to an increase in the rate of response but also in the percentage of patients developing high grade toxicity [6].

The CheckMate 067 study randomized 945 patients with unresectable or stage IV melanoma in a 1:1:1 ratio to treatment with ipilimumab monotherapy, nivolumab monotherapy or ipilimumab plus nivolumab [6]. With 36 months of minimum follow-up, the response rate following ipilimumab, nivolumab, or combination therapy was 19%, 44%, and 58%, respectively. However, grade 3 or 4 treatment related adverse events developed in 28%, 21%, and 59% of ipilimumab, nivolumab and combination therapy patients, respectively. Any grade and grade 3 or 4 diarrhea developed in 34% and 6% of ipilimumab treated patients, 21% and 3% of nivolumab treated patients, and 45% and 9% of combination therapy treated patients. Any grade and grade 3 or 4 colitis developed in 11% and 8% of ipilimumab treated patients, 2% and 1% of nivolumab treated patients, and 13% and 8% of combination therapy treated patients. Discontinuation of immunotherapy due to treatment-related grade 3–4 diarrhea/colitis was required in 12.2%, 2.5% and 16.6% of these patients, respectively [6].

In a retrospective observational study of 103 melanoma patients treated with ipilimumab, 30 patients (29%) developed diarrhea with 23 patients (22%) developing severe diarrhea/colitis requiring systemic corticosteroid therapy [7]. Infliximab, an anti-TNF-α-therapy, was used as rescue therapy in steroid refractory patients.

Several studies of exploratory biomarkers for identifying immune-related gastrointestinal adverse events have been reported to date [8–13]. However, interactions between cells circulating in the blood with peripheral tissues might alter blood cell gene expression, thereby conferring information about physiologic disruptions in various tissue compartments. We previously identified and validated a response-predictive gene signature derived from the peripheral blood mRNA expression of 15 genes prior to treatment of melanoma patients with tremelimumab [14]. Given the ability for peripheral gene expression signatures to predict response, we considered the possibility that pre-treatment or early on-treatment peripheral blood gene expression might also predict for toxicity to tremelimumab [8].

An unmet clinical need exists for a biomarker to predict immune-related diarrhea/colitis prior to onset. Using two large clinical trial datasets, we tested whether peripheral blood gene signatures could predict CLTA-4 blockade-associated diarrhea.

## Methods

### Patient population

The patient population in the current analysis is derived from two large independent clinical trials exploring the efficacy of tremelimumab in patients with advanced melanoma. Both of these trials have been previously described in detail [2, 14–16]. The phase II trial served as the discovery dataset, and the phase III trial served as the validation dataset. Patients enrolled in each study received 15 mg/kg tremelimumab every 90 days. Both clinical studies were conducted on a worldwide basis, including patients from 19 countries in North America, Europe and Australia. The patients in the discovery dataset were chemotherapy-refractory, while the patients in the validation dataset were treatment-naïve. One year overall survival data for patients enrolled in the phase II and III studies as a whole and in the biomarker study population is shown in Table 1. Clinical and demographic information was obtained and recorded by authorized personnel after obtaining written informed consent. Protocols and consent forms were approved by the local institutional review boards.

**Table 1:** Comparison of patient characteristics in the full study populations and the biomarker study eligible populations

	Discovery		Validation	
	Full study population	Biomarker population	Full study population	Biomarker population
Number of patients	250	150	328	210
Age, median (range) years	53 (18–89)	53 (18–89)	57 (22–90)	59 (22–90)
Gender, n (%)				
Male	151 (60%)	94 (63%)	190 (58%)	117 (56%)
Female	99 (40%)	56 (37%)	138 (42%)	93 (44%)
Objective Response, n (%)				
Partial response	16 (7%)	20 (13%)	36 (11%)	28 (13%)
Stable disease or progressive disease	235 (93%)	130 (87%)	292 (89%)	182 (87%)
Grade of diarrhea				
Any grade	99 (39%)	60 (40%)	166 (51%)	92 (44%)
Grade 2–4	50 (20%)	21 (14%)	89 (27%)	56 (27%)
Grade 3–4	37 (15%)	9 (6%)	60 (18%)	27 (13%)
Prior chemotherapy	Yes	Yes	No	No
One year survival n (%)				
Patient alive at one year	100 (40%)	43 (29%)	170 (52%)	118 (56%)
Patient deceased at one year	150 (60%)	107 (71%)	158 (48%)	92 (44%)
Stages of Disease				
IIIB and IIIC	8 (3%)	5 (3%)	20 (6%)	13 (6%)
IV M1A	27 (11%)	16 (11%)	46 (14%)	35 (17%)
IV M1B	52 (21%)	30 (20%)	75 (23%)	49 (23%)
IV M1C	164 (65%)	99 (66%)	187 (57%)	113 (54%)
Live in United States n (%)				
U.S.	114 (46%)	62 (41%)	62 (19%)	44 (21%)
Non-U.S.	136 (54%)	88 (59%)	266 (81%)	166 (795)

The primary objective of this retrospective analysis was to correlate peripheral blood gene expression signatures with the development of grade 2–4 diarrhea. We focused on the distinction between grade 0–1 versus grade 2–4 diarrhea, reasoning that many cases of grade 1 diarrhea are not treatment-related and grade 2–4 diarrhea requires closer monitoring, intervention, and/or discontinuation of therapy. Although grade 2 diarrhea does not always need treatment with high dose steroids consensus recommendations for toxicity management recommend holding treatment and initiation of steroids if no improvement to grade 1 in several days. Only patients with both pre- and 30-day post-treatment blood samples and clinical data capturing worst case diarrhea were included. Due to the nature of the data transfer agreement, data regarding the presence and severity (grade 1–5) of diarrhea were available but not the time of onset, treatment, or resolution. Grading of toxicity was as defined by the National Cancer Institute Common Terminology Criteria for Adverse Events version 3.0.

### Whole-blood gene expression profiling

A set of 169 genes related to cancer, immunity, CTLA-4 pathway, and inflammation was analyzed from pre-treatment, and 30-day-post-treatment, peripheral blood specimens from the discovery and validation cohorts (Additional file 1: Table S1). This set of genes included a 72-gene inflammatory panel consisting of genes selected from literature review of studies of inflammation [17]. Our approach to gene selection has previously been described [14, 16]. Whole-blood samples (2.5 ml) were collected into PAXgene™ RNA stabilization tubes (PreAnalytiX, Hombrechtikon, CH). RNA was extracted from the samples using a PAXgene™ Blood RNA Kit (PreAnalytiX) in accordance with the manufacturer’s protocol. The quality of the RNA was verified on an Agilent® 2100 Bioanalyzer (Agilent Technologies, Palo Alto, CA), and the quantity of RNA was determined by NanoDrop® ND-1000 spectrophotometer (Thermo Scientific, Wilmington, DE). First-strand complementary DNA was synthesized from random hexamer-primed RNA templates using TaqMan® reverse-transcription reagents. Individual target-gene amplification was multiplexed with 18S rRNA endogenous control and run in triplicate in 384-well format on 7900HT fast real-time PCR systems. Primers/probes were custom designed so that the amplification efficiency was within 90%. For quality control, all replicate cycle threshold (*C*T) values (both target gene and endogenous control) were independently checked and automatically filtered by rule. Normalized *C*T values (Δ*C*t) for each amplified target gene replicated were calculated. Resulting triplicate Δ*C*t values for individual target genes were averaged, yielding a final Δ*C*t value.

### Statistical analysis

With the 169-gene panel, we first analyzed the expression of individual genes in the discovery dataset for their ability to distinguish grade 0–1 versus grade 2–4 diarrhea (t-test). This process was repeated for both the baseline (pre-treatment) and 30-day post-treatment gene expression data. We followed the previously-described statistical analysis methodology on the 30-day post-treatment blood samples as used in the development and validation of gene expression whole-blood RNA predictive response and survival signatures for advanced melanoma patients treated with tremelimumab [14]. Synergistic gene pairs, or “2-gene core models,” were trained to distinguish grade 0–1 versus grade 2–4 diarrhea in the post-treatment discovery dataset (*N* = 150) using the Statistical Innovations (Belmont, MA) CORExpress 1.1 commercial software package. Approximately 120 post-treatment 2-gene core models significantly predicted grade 0–1 versus grade 2–4 diarrhea in the discovery dataset and were successfully validated in the validation dataset (*N* = 210). Larger, post-treatment classifier models were then constructed in the discovery dataset by combining validated 2-gene core models using the step-down, 3-components, logistical regression functions. An optimal 16-gene signature was developed in the discovery dataset with a defined cut-off. Six of the 16 genes were classified as predictor genes and 10 genes were classified as enhancer genes. Enhancer genes and their value have been previously described [14, 18] The 16-gene signature AUC was checked with publically-available MedCalc version 17 ROC analysis and *p*-value software. (MedCalc Software, Ostend, Belgium). The 16-gene signature was then validated in the validation dataset using the same cut-off as in the discovery dataset.

## Results

**Fig. 1:**
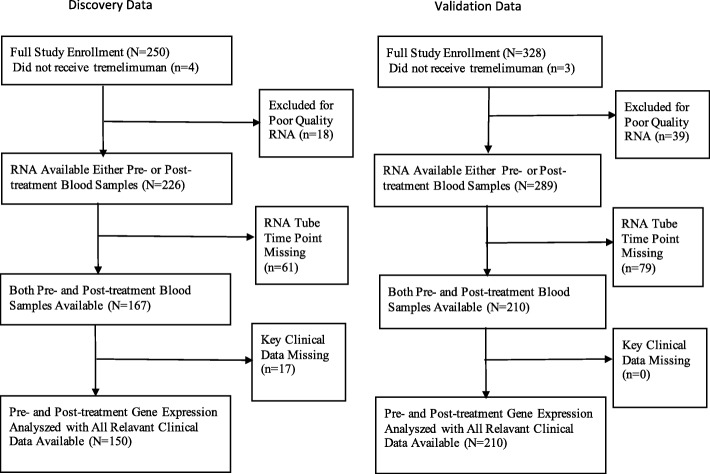
Main clinical trial enrollment and study population exclusions

Among the 251 patients enrolled in the phase II trial comprising the discovery cohort, 150 patients had both pre- and post-treatment peripheral blood gene expression data and clinical data regarding diarrhea. Among the 328 patients enrolled in the tremelimumab arm of the phase III trial comprising the validation cohort, 210 patients had both pre- and post-treatment peripheral blood gene expression data and clinical data regarding diarrhea. The reasons for patient exclusion are detailed in Fig. 1. The base-line and post-treatment characteristics of the full study populations, and the populations included in our analysis were very similar, as detailed in Table 1.

In the discovery cohort, 21 of 150 patients (14%) developed grade ≥ 2 diarrhea, and in the validation biomarker cohort, 56 of 210 patients (27%) developed grade ≥ 2 diarrhea.

**Fig. 2:**
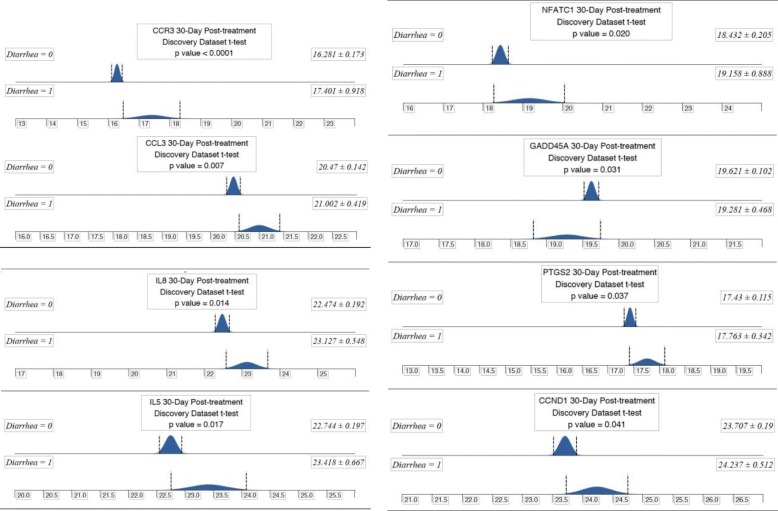
Graphic presentation of the difference in expression of each of the eight genes with statistical significance (defined by *p* value < 0.05) in discriminating patients who develop grades 0–1 versus 2–4 diarrhea. T-test analysis performed on blood samples obtained 30 days post initiation of tremelimumab treatment

Pre-treatment peripheral blood from the discovery dataset (*n* = 150) revealed no individual gene that was significantly predictive of the development of grade 2 or higher diarrhea/colitis (*p* < 0.05)**.** Therefore we performed no further testing on the pre-treatment blood samples. Post-treatment blood samples, however, did reveal eight genes of significance discriminating grade 0–1 versus grade 2–4 diarrhea (CCR3 *p* = 0.0001, CCL3 *p* = 0.007, IL8 *p* = 0.014, IL5 *p* = 0.017, NFATC1 *p* = 0.020, GADD45A *p* = 0.031, PTGS2 *p* = 0.037, CCND1 *p* = 0.041) as depicted in Fig. 2. In all 8 cases gene expression in the patients with grade 0–1 diarrhea are tightly grouped while those with grade 2–4 patients more widely diverse and clearly differentiated for the Grade 0–1. Thus, further analysis was performed on the 30-day post-treatment blood samples to identify a gene signature predictive for the development of grade 2–4 diarrhea.

Using the 30 day post-treatment discovery dataset, we trained synergistic gene pairs to distinguish between grade 0–1 and grade 2–4 diarrhea. We identified 120 post-treatment 2-gene core models which significantly predicted grade 0–1 versus grade 2–4 diarrhea and which were subsequently successfully validated in the validation dataset (*N* = 210).

In the discovery dataset, construction of larger classifier models identified a 16-gene signature which discriminated the 129 patients who experienced grade 0–1 diarrhea from the 21 patients who experienced grade 2–4 diarrhea with an AUC of 0.814 (95% CI 0.743, 0.873; *p* < 0.0001). The 16-gene signature is represented by the formula:

$$ >\hbox{-} 26.17\hbox{-} \left(\mathrm{CARD}{12}^{\ast }0.351\right)+\left(\mathrm{CCL}{3}^{\ast }0.833\right)+\left(\mathrm{CCR}{3}^{\ast }0.774\right)+\left(\mathrm{CXCL}{1}^{\ast }0.166\right)\hbox{-} \left(\mathrm{F}{5}^{\ast }0.319\right)+\left(\mathrm{F}\mathrm{AM}210{\mathrm{B}}^{\ast }0.851\right)\hbox{-} \left(\mathrm{GADD}45{\mathrm{A}}^{\ast }0.605\right)\hbox{-} \left(\mathrm{IL}18{\mathrm{bp}}^{\ast }0.423\right)\hbox{-} \left(\mathrm{IL}2{\mathrm{RA}}^{\ast }0.350\right)+\left(\mathrm{IL}{5}^{\ast }0.378\right)+\left(\mathrm{IL}{8}^{\ast }0.184\right)\hbox{-} \left(\mathrm{MMP}{9}^{\ast }0.126\right)+\left(\mathrm{PTGS}{2}^{\ast }0.681\right)\hbox{-} \left(\mathrm{SOCS}{3}^{\ast }0.212\right)+\left(\mathrm{TLR}{9}^{\ast }0.438\right)\hbox{-} \left(\mathrm{UBE}2{\mathrm{C}}^{\ast }0.63\right) $$

Although NFATC1 and CCND1 were predictors as single genes when 2-gene combinations were trained NFATC1 and CCND1 dropped out of the algorithm as other 2-gene combinations were more powerful in predicting Grades 2–4 diarrhea. This may be because NFATC1 and CCND1 2-gene combinations did not have as strong enhancer/suppressor support as the other six predictors. The six predictor genes included in the signature are CCR3, CCL3, IL8, IL5, GADD45, and PTGS2. The remaining 10 genes included in the signature are suppressors which, while not predictive in their own right, enhance the predictiveness of the six predictive genes.

**Fig. 3:**
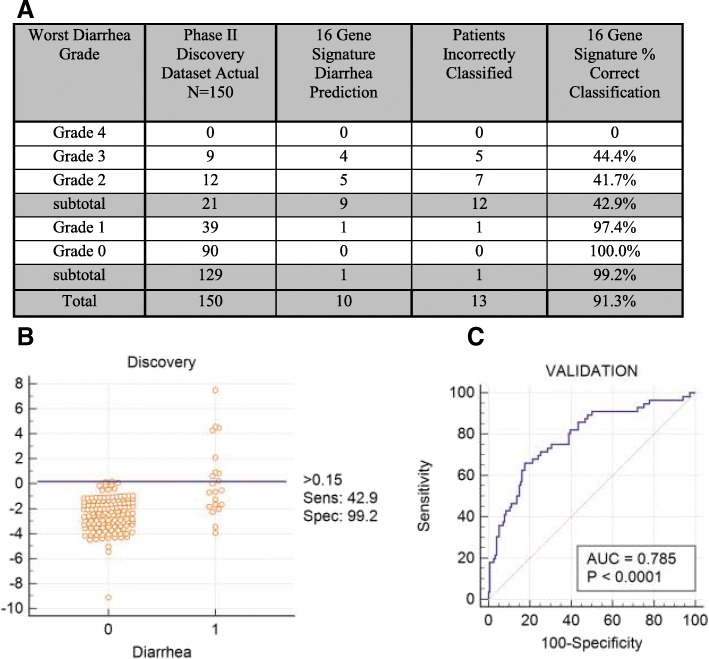
The 16-gene signature discriminates in the 30-days post-treatment discovery dataset the 129 patients who experienced grade 0–1 from the 21 patients who experienced grade 2–4 diarrhea with an AUC of 0.814 (95% CI 0.743, 0.873; *p* < 0.0001). **a** Accuracy of patient classification of as a function of diarrhea grade using the 16 gene signature. **b** Dot diagram depicting the 16-gene signature results in discovery dataset patients (*N* = 150) experiencing grade 0–1 diarrhea (designated as 0) versus grade 2–4 diarrhea patients (designated as 1) with a 0.15 cut-off. **c** Receiver operating characteristic curve plotting sensitivity of the 16-gene signature on the y axis and 100-specificity on the x axis in discovery dataset patients

A risk score cutoff equal to 0.15 for the 16-gene signature (positive score predicts grade 2–4 diarrhea) was associated with sensitivity 42.9%, specificity 99.2%, PPV 90.0%, NPV 91.4% and correct classification of 91.3% of the patients (See - Fig. 3). In Table 2 the mean pre- and post- treatment expression values for each of the 16 signature genes is listed demonstrating the relative statistical significance of change in individual gene expression when comparing patients who developed grade 0–1 diarrhea to those who developed grade 2–4 diarrhea. Differences in pre-treatment and post-treatment expression of each of the 169 genes tested between patients who developed grade 0–1 versus grade 2–4 diarrhea is shown in Additional file 2: Table S2.

**Table 2:** Comparison of mean delta Ct values for each of the 16 genes in the 16 gene signature in discovery dataset patients pre-treatment and post-treatment. Expression of the genes in patients who developed grade 0–1 diarrhea are compared to patients who developed grade 2–4 diarrhea

Discovery Dataset							
	Gene Expression Delta Ct Values Pre-Treatment Means			Gene Expression Delta Ct Values Post-Treatment Means			
	Grade 0–1 Diarrhea Pre Treatment *N* = 129	Grade 2–4 Diarrhea Pre Treatment *N* = 21	Pre-Treatment Difference	Grade 0–1 Diarrhea Post Treatment *N* = 129	Grade 2–4 Diarrhea Post Treatment *N* = 21	Post Treatment Difference	Post Treatment Difference *p* value
Predictor Gene Name							
CCR3	16.71	16.98	0.27	16.28	17.40	1.12	0.0001
CCL3	20.60	20.90	0.30	20.47	21.00	0.53	0.007
IL8	22.14	21.84	−0.30	22.47	23.13	0.65	0.014
IL5	23.08	23.20	0.12	22.74	23.42	0.67	0.017
GADD45A	19.86	20.05	0.19	19.62	19.28	−0.34	0.031
PTGS2	17.40	17.49	0.09	17.43	17.76	0.33	0.037
Enhancer Variable Gene Name							
MMP9	14.29	14.41	0.11	14.20	13.69	−0.52	0.078
CARD12	17.63	17.73	0.09	17.54	17.25	−0.29	0.096
SOCS3	18.31	18.42	0.11	18.11	17.78	−0.33	0.114
F5	17.98	18.04	0.06	17.69	17.40	−0.29	0.120
TLR9	18.14	18.11	−0.03	18.01	18.14	0.14	0.270
FAM210B	15.21	15.32	0.11	15.35	15.58	0.23	0.325
IL18BP	17.55	17.42	−0.13	17.32	17.46	0.14	0.355
UBE2C	21.23	21.31	0.08	20.31	20.17	−0.14	0.358
CXCL1	19.80	19.87	0.07	19.95	20.09	0.14	0.381
IL2RA	19.04	18.84	−0.20	18.44	18.57	0.12	0.486

### Gene signature validation

**Fig. 4:**
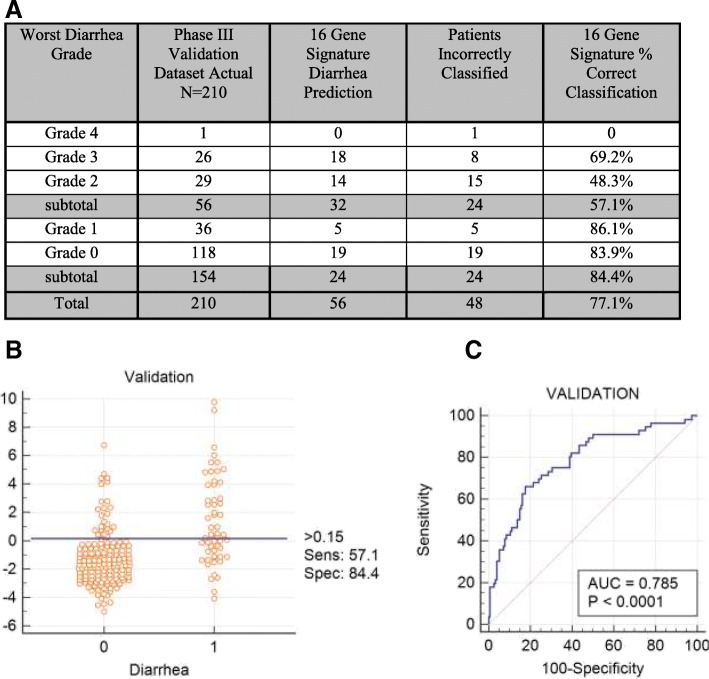
The 16-gene signature discriminates in the validation dataset the 154 patients who experienced grade 0–1 from the 56 patients who experienced grade 2–4 diarrhea with an AUC 0.785 (95% CI 0.723, 0.838; *p* < 0.0001). **a** Accuracy of patient classification of as a function of diarrhea grade using the 16 gene signature. **b** Dot diagram depicting the 16-gene signature results in discovery dataset patients (N = 150) experiencing grade 0–1 diarrhea (designated as 0) versus grade 2–4 diarrhea patients (designated as 1) with a 0.15 cut-off. **c** Receiver operating characteristic curve plotting sensitivity of the 16-gene signature on the y axis and 100-specificity on the x axis in validation dataset patients

The 16-gene signature was tested in the validation dataset and shown to discriminate the 154 patients who experienced grade 0–1 diarrhea from the 56 patients who experienced grade 2–4 diarrhea with an AUC 0.785 (95% CI 0.723, 0.838; *p* < 0.0001). The same diarrhea risk score cutoff of 0.15 utilized in the discovery dataset was applied in the validation dataset, revealing a sensitivity 57.1%, specificity 84.4%, PPV 57.1%, NPV 84.4% and correct classification of 77.1% of the patients. (See Fig. 4.)

The development of grade 2 diarrhea as the worst grade toxicity does not necessarily lead to permanent discontinuation of treatment. Therefore, we also tested the ability of the 16-gene signature to differentiate grades 0–2 versus grade 3–4 diarrhea. A risk score cutoff equal to − 0.20 for the 16-gene signature was associated with an AUC of 0.812, (95% CI 0.74 to 0.87, *p* = 0.0007), sensitivity 55.6%, specificity 94.3%, PPV 38.5%, NPV 97.1% and correct classification of 92.0% of the patients. The same diarrhea risk score cutoff of − 0.20 utilized in the discovery dataset was applied in the validation dataset, revealing an AUC of 0.814, (95% CI 0.76 to 0.86, *p* < 0.0001), a sensitivity 74.1%, specificity 78.1, PPV 33.3%, NPV 95.3% and correct classification of 77.6% of the patients.

A risk cut off score of 0.15 to differentiate grades 0–1 versus grade 2–4 diarrhea was chosen to maximize positive and negative predictive values but the only patient with stage 4 diarrhea had a predictive score of − 1.685 and therefore not correctly classified. Choosing a risk cut off of − 1.70 would allow for correct classification of the patient with grade 4 diarrhea. Using this cut off when testing the 16-gene signature on the discovery data set showed a 66.7% sensitivity, 74.4% specificity, 29.8% PPV, and 93.2% NPV with correct classification of 73.3% of patients (Additional file 3: Table S3a). Applying this cut off to the validation data set revealed an 89.3% sensitivity, 51.9% specificity, 40.3% PPV, and 93% NPV with correct classification of 61.9% of patients (Additional file 3: Table S3b).

## Discussion

This study demonstrates that changes in the expression of genes in the whole blood associate with the development of a clinically-relevant irAE in a cohort of patients treated with immune checkpoint blockade. Using a discovery dataset comprised of blood samples derived from tremelimumab treated melanoma patients, we identified a 16 gene signature that may distinguish the onset of severe versus mild or no diarrhea. The signature was further characterized in a validation dataset derived from a later tremelimumab clinical trial. The study focused on diarrhea/colitis irAEs but supports the hypothesis that altered gene expression in peripheral blood can identify biomarkers predictive of other types of immune mediated toxicity. Strengths of our study include utilization of two large clinical trial datasets exploring CTLA-4 blockade in patients with advanced melanoma, collection of blood specimens prospectively in tubes optimized for mRNA preservation, the uniform follow-up of patients with clinical-trial level adverse event data capture, and the derivation of the biomarker from blood samples as opposed to more difficult to obtain colonic or tumor tissue biopsies.

In our study, the 16-gene signature validated successfully with PPV 90.0%, NPV 91.4% and correct classification of 91.3% of the patients in the discovery dataset and PPV 57.1%, NPV 84.9% and correct classification of 77.1% of the patients in the validation dataset. This provides proof of concept that changes in gene expression in peripheral blood (as opposed to gene expression in tumor tissue or the organ involved directly by the toxicity) can predict for immune mediated toxicity following treatment with a CTLA-4 inhibitor. Differences in the NPV and PPV seen in the discovery and validation datasets may reflect differences in patient characteristics in the two studies. The discovery dataset encompassed patients who had previously been treated with chemotherapy while patients included in the validation dataset were treatment naïve. It is possible that prior chemotherapy treatment induces lymphopenia and decreases the inflammatory activity of the immune system which may explain the lower rate of grade 2 or greater GI toxicity in the discovery dataset when compared to the validation dataset. We were not able to investigate the ability of gene expression changes to predict for the development of other types of immune mediated toxicity as we do not have access to patient specific toxicity data for other toxicities.

While we were able to validate a predictive gene signature based on mRNA expression in 30-day post-treatment blood specimens, we were not able to identify a predictive gene signature when using pre-treatment blood samples. However, an exploratory analysis of 35 melanoma patients treated with neoadjuvant ipilimumab demonstrated a significant association of baseline circulating IL-17 levels and the subsequent development of severe diarrhea/colitis [12]. The predictive genes in the signature we identified are not proven to regulate IL-17 expression or activity. However IL-17 is known to regulate the expression of the differentially expressed gene IL-8 [19]. It would be interesting to compare the predictiveness of our gene signature to baseline IL-17 expression, but unfortunately we do not have access to baseline IL-17 expression data in patients treated in the phase II and III tremelimumab studies. We hypothesize that modulation of the immune system induced by the immune checkpoint blockade results in broader gene expression changes in the blood that can be detected prior to the clinical onset of severe diarrhea but are dependent on prior immunotherapy treatment.

Two main limitations of our study are that (1) the immunotherapy tested was tremelimumab rather than the FDA-approved ipilimumab; and (2) we had access to data only regarding the presence and severity of diarrhea in each patient but not the timing of onset, treatment, and/or duration the CTLA-4 blockade immunotherapy. While developing high grade diarrhea within the first 30 days following the initial dose of tremelimumab is possible, the number of patients doing so is expected to be very low as the average time to develop severe gastrointestinal irAEs following anti-CTLA-4 treatment is three infusions [3, 20].

A post-treatment biomarker of immune-related diarrhea would not inform decisions regarding initiation of immune checkpoint blockade in potentially at-risk patients. Nevertheless, identifying patients likely to develop high grade diarrhea prior to development of the adverse event would allow for closer monitoring and consideration of early intervention, which can be of substantial benefit to patient safety while controlling healthcare costs. A limitation is that with the median time to onset of diarrhea being 6–7 weeks a biomarker based on expression of genes in a day 30 blood sample plus time to perform the gene expression analysis limits the ability of the signature to serve predictively. Unfortunately we do not have blood samples at earlier time points post treatment to test for predictive expression changes. However the association of high grade diarrhea with changes in expression of a specific signature of genes in peripheral blood is proof of concept. The gene signature can provide mechanistic insights into early on-treatment effects on gene expression.

Different immunotherapy regimens are likely to alter peripheral blood gene expression differently. The CTLA-4 inhibitor ipilimumab which is FDA approved for treatment of stage IV melanoma is an IgG1 antibody while tremelimumab is IgG2. Treatment with anti-PD-1 monotherapy or combined anti-CTLA-4 plus anti-PD-1 blockade modulates the immune system differently than does anti-CTLA-4 monotherapy. The identification of the 16-gene signature in the context of tremelimumab treatment supports the idea of searching for pre- or early on treatment blood based toxicity biomarkers based on changes in gene expression. Given that tremelimumab modulates the immune system differently than currently FDA approved immune checkpoint inhibitors (in terms of mechanism of action and antibody isotype) it is very likely that the predictive value of the16 gene signature will be different in the context of these treatments and that different predictive gene signatures will be identified as optimal.

The 16 gene signature we identified contains six genes which are predictive. The remaining 10 genes are suppressors which do not individually predict for the development of diarrhea but enhance the predictive ability of the predictors. Understanding the mechanism of action of genes included in the identified signature can provide clues into mechanisms facilitating the development of immune-mediated diarrhea. Identifying the function of the predictive genes and their association with the intestinal tract can help in understanding the cellular processes driving CTLA-4 blockade-related diarrhea.

In the discovery dataset, the mean expression of five of the predictor genes was upregulated 1 to 2.2 fold in patients who developed grade 2–4 diarrhea when compared to patients who did not. The up-regulated genes included CCL3 (cytokine), CCR3 (chemokine receptor), IL5 (cytokine), IL8 (chemokine), PTGS2 (cyclooxygenase-2 or COX-2), all of which are involved with immune response to inflammation. This suggests that peripheral down regulation of these immune response genes might be indicative of the clinical onset of severe diarrhea.

Of the six predictive genes associated with this gene signature, three genes – IL-8, CCR3, and CCL3 – have a known association with cases of diarrhea induced by etiologies other than checkpoint inhibitors. These genes are all involved with the mobilization of inflammatory cells to the gut mucosa leading to the breakdown of the intestinal barrier. IL-8, a chemokine, is critical for the chemotaxis of polymorphonuclear leukocytes in the setting of gastrointestinal infections leading to breakdown of the epithelial barrier [21]. CCL3, a cytokine, is involved with T cell recruitment in inflammatory disease and plays a central role in eospinphilic trafficking into the gastrointestinal tract [22]. Blocking expression of this protein in mice with a CCL3 binding protein decreased expression of CD4+ and CD8+ T cells infiltrating the small intestine leading to a reduction in inflammatory infiltrate.. In mouse models of food allergen-induced GI eosinophilic inflammation, blocking expression of CCR3 significantly reduced the severity of diarrhea, eosinophilic inflammation, and mucosal injury [23].

All three of these genes, in addition to the predictive genes PTGS2 and IL5, were downregulated in the peripheral blood of patients who experienced grade 2–4 diarrhea. As the predictive signature is based on mRNA expression in peripheral blood we cannot determine whether gene expression and respective translated protein is up or down regulated in the actual colonic microenvironment. It is possible that cells producing these cytokines have moved from the periphery into the colonic mucosa and submucosa, leading to lower gene expression in peripheral blood and to the development of diarrhea.

While not individually predictive of diarrhea induced by tremelimumab, two of the enhancer variable genes in the gene signature also appear to be associated with diarrhea from other etiologies. CXCL1 is upregulated in the colonic mucosa in 5-FU-induced diarrhea and pre-clinic data has shown that inhibition of CXCL1 may prevent 5-FU-induced diarrhea from developing [24]. In colitis associated with inflammatory bowel disease, epithelial and fecal MMP-9 levels correlate with severity of colitis [25].

Despite this gene signature being predictive for diarrhea, three of the enhancer variable genes appear to either directly or indirectly have a protective effect against diarrhea in other settings. TLR9 protects against intestinal epithelium damage and thus preventing some forms of diarrhea [26]. Loss of IL-18 bp expression is associated with severe colitis and loss of mature goblet cells in mice [27]. IL-18 bp is a negative regulator of IL-18, a gene critical in driving intestinal barrier breakdown leading to colitis. SOCS3, a key intracellular regulator of signaling by granulocyte colony-stimulating factor, has a protective effect against graft-versus-host disease related diarrhea after allogeneic transplant [28].

A toxicity predictive biomarker could potentially be used to enrich the selection of patients for clinical trials exploring strategies to mitigate the toxicity. In an unselected melanoma patient population treated with ipilimumab, prophylactic budesonide did not demonstrate efficacy in terms of decreasing diarrhea risk [29]. A similarly designed clinical trial in which a prophylactic strategy administered only to patients with a biomarker-defined high likelihood of developing grade 2–4 diarrhea/colitis would provide an enriched patient population to assess the efficacy of preventative strategies.

## Conclusions

Our study demonstrates that whole blood samples can be used to identify gene expression signatures that differentiate patients who develop grade 2 or higher diarrhea or colitis from those who develop mild or no diarrhea following the initiation of treatment with tremelimumab. We do not validate a signature that identifies all patients who will develop diarrhea severe enough to require treatment discontinuation. However, our data suggests that this minimally invasive strategy can identify early in a given patient’s treatment course those at risk for the development of high grade diarrhea or colitis. This information potentially could allow for early intervention strategies that would limit irAE severity or prevent toxicity development and thereby extend treatment duration. While anti-CTLA-4, anti-PD-1, and anti-PD-L1 inhibitors have shown significant efficacy as treatment in a range of malignancies, the continued long term use of these immunotherapy treatments in a subset of patients is limited by the development of high grade immune mediated toxicity. As such, the development of biomarkers predictive for the development of high grade toxicity is an unmet clinical need. It is likely that toxicity developed in the context of anti-PD-1 or anti-PD-L1 immunotherapy or other CTLA-4 inhibitors will not occur through identical mechanisms, and therefore predictive gene signatures will differ depending on the specific immune therapy. Our data suggests that biomarker identification using a whole blood transcriptome approach is technically feasible and should be explored in the context of additional gene panels and time points, other blood based biomarkers, and other immune mediated toxicities.

## Additional files

Additional file 1: **Table S1** List of Genes with Full Name and Aliases of Each. (DOCX 27 kb)

Additional file 2: **Table S2** Differences in pre-treatment and post-treatment expression of each of the 169 genes tested between patients who developed grade 0–1 versus grade 2–4 diarrhea. (a) discovery dataset and (b) validation dataset. (DOCX 17 kb)

Additional file 3: **Table S3 (a)** Using a − 1.70 cut off when testing the 16-gene signature on the discovery data set showed a 66.7% sensitivity, 74.4% specificity, 29.8% PPV, and 93.2% NPV with correct classification of 73.3% of patients. Accuracy of patient classification of as a function of diarrhea grade using the 16-gene signature. **(b)** Applying this − 1.70 cut off to the validation data set revealed an 89.3% sensitivity, 51.9% specificity, 40.3% PPV, and 93% NPV with correct classification of 61.9% of patients. Accuracy of patient classification of as a function of diarrhea grade using the 16-gene signature. (DOCX 92 kb)

## Abbreviations

AUC: Area under the curve
Ct: Cycle threshold
CTLA-4: Cytotoxic T-lymphocyte associated protein 4
FDA: Food and drug administration
IHC: Immunohistochemistry
N: Number
NPV: Negative predictive value
PCR: Polymerase chain reaction
PD-1: Programmed death-1
PD-L1: Programmed death-ligand 1
RNA: Ribonucleic acid
rRNA: Ribosomal ribonucleic acid
Sens: Sensitivity
Spec: Specificity
