# Supplementary material for: A whole-blood RNA transcript-based gene signature is associated with the development of CTLA-4 blockade-related diarrhea in patients with advanced melanoma treated with the checkpoint inhibitor tremelimumab
Source: J Immunother Cancer. 2018 Sep 18;6:90. doi: 10.1186/s40425-018-0408-9 (PMC6145108; doi:10.1186/s40425-018-0408-9)
Supplement: Supplementary file 1 — Table S1 List of Genes with Full Name and Aliases of Each. (DOCX 27 kb) [file 40425_2018_408_MOESM1_ESM.docx]

List of Genes with Full Name and Aliases of Each

| **Gene** | **Full Name** | **Aliases** |
| --- | --- | --- |
| **ADAM17** | ADAM Metallopeptidase Domain 17 | ADAM18, CD156B, NISBD1, NISBD, TACE, CSVP |
| **ALOX5** | Arachidonate 5-Lipoxygenase | 5-LOX, 5LPG, LOX-5, 5-LO, LOG5, 5-lipoxygenase |
| **ANLN** | Anillin, Actin Binding Protein | FSGS8, Scraps, scra |
| **APAF1** | Apoptotic Peptidase Activating Factor 1 | CED4, APAF-1, KIAA0413 |
| **AXIN2** | Axin 2 | conductin, axil, axin-2, ODCRCS |
| **BAD** | BCL2-Associated Agonist Of Cell Death | BBC2, bcl2-L-8, BCL2L8, BBC6 |
| **BAX** | BCL2-Associated X Protein | Baxdelta2G9, Baxdelta2G9omega, Baxdelta2omega, bcl2-L-4, BCL2L4 |
| **BLVRB** | Biliverdin Reductase B | FR, GHBP, HEL-S-10, SDR43U1, BVRB, FLR, BVR-B |
| **BPGM** | 2,3-Bisphosphoglycerate Mutase | DPGM |
| **BRCA1** | Breast Cancer 1, Early Onset | BRCC1, BRCAI, FANCS, IRIS, PPP1R53, BROVCA1, PNCA4, PSCP, RNF53 |
| **C1QA** | Complement Component 1, Q Subcomponent, A Chain |  |
| **CASP1** | Caspase 1, Apoptosis-Related Cysteine Peptidase | p45, caspase-1, beta, convertase, IL1B-convertase, IL1BC, ICE, CASP-1, IL-1BC, IL1BCE |
| **CASP3** | Caspase 3, Apoptosis-Related Cysteine Peptidase | CPP32B, apopain, caspase-3, procaspase3, CPP32, CASP-3, CPP-32, SCA-1 |
| **CCL3** | Chemokine (C-C Motif) Ligand 3 | LD78ALPHA, SCYA3, MIP1A, G0S19-1, MIP-1-alpha, SIS-beta |
| **CCL5** | Chemokine (C-C Motif) Ligand 5 | SIS-delta, RANTES, SISd, eoCP, D17S136E, SCYA5, TCP228 |
| **CCND1** | Cyclin D1 | U21B31, D11S287E, BCL1, PRAD1, BCL-1 |
| **CCR3** | Chemokine (C-C Motif) Receptor 3 | CD193, CMKBR3, CKR3, CC-CKR-3, CCR-3 |
| **CCR5** | Chemokine (C-C Motif) Receptor 5 (Gene/Pseudogene) | ChemR13, CD195, CKR-5, CKR5, CCCKR5, IDDM22, CMKBR5, CC-CKR-5, CCR-5 |
| **CCR7** | Chemokine (C-C Motif) Receptor 7 | CD197, CMKBR7, EBI1, BLR2, CC-CKR-7, CCR-7, CDw197, EVI1 |
| **CCR9** | Chemokine (C-C Motif) Receptor 9 | CDw199, GPR28, CC-CKR-9, GPR-9-6, CCR-9 |
| **CD19** | CD19 Molecule | B4, CVID3 |
| **CD28** | CD28 Molecule | Tp44 |
| **CD4** | CD4 Molecule | CD4mut |
| **CD40** | CD40 Molecule, TNF Receptor Superfamily Member 5 | P50, CDW40, TNFRSF5, Bp50 |
| **CD40LG** | CD40 Ligand | TRAP, CD154, T-BAM, gp39, hCD40L, IMD3, IGM, HIGM1, TNFSF5, CD40-L, CD40L |
| **CD80** | CD80 Molecule | B7-1, B7.1, CD28LG1, CD28LG, LAB7, B7, BB1 |
| **CD86** | CD86 Molecule | B7-2, B7.2, LAB72, CD28LG2, B70, BU63, FUN-1 |
| **CD8A** | CD8a Molecule | Leu2, p32, CD8, MAL |
| **CDC25A** | Cell Division Cycle 25A | CDC25A2 |
| **CDH1** | Cadherin 1, Type 1, E-Cadherin (Epithelial) | E-Cadherin, Arc-1, CD324, cadherin-1, uvomorulin, ECAD, LCAM, UVO, CDHE, uvomorulin) |
| **CDK2** | Cyclin-Dependent Kinase 2 | CDKN2, p33(CDK2) |
| **CDKN1A** | Cyclin-Dependent Kinase Inhibitor 1A (P21, Cip1) | CIP1, P21, p21CIP1, CDKN1, WAF1, CAP20, MDA-6, SDI1, MDA6, PIC1 |
| **CDKN1B** | Cyclin-Dependent Kinase Inhibitor 1B (P27, Kip1) | MEN1B, P27KIP1, CDKN4, MEN4, KIP1 |
| **CDKN2A** | Cyclin-Dependent Kinase Inhibitor 2A | CDK4I, p19, INK4, INK4A, P14, P14ARF, P16-INK4A, P16INK4, P16INK4A, P19ARF, TP16, CDKN2, CMM2, P16, MLM, MTS1, ARF, MTS-1, p16-INK4 |
| **CDKN2D** | Cyclin-Dependent Kinase Inhibitor 2D (P19, Inhibits CDK4) | INK4D, p19, p19-INK4D |
| **CHPT1** | Choline Phosphotransferase 1 | CPT, CPT1, hCPT1 |
| **CIITA** | Class II, Major Histocompatibility Complex, Transactivator | CIITAIV, NLRA, C2TA, MHC2TA |
| **CNKSR2** | Connector Enhancer Of Kinase Suppressor Of Ras 2 | MAGUIN, CNK2, KSR2, KIAA0902 |
| **CSF2** | Colony Stimulating Factor 2 (Granulocyte-Macrophage) | sargramostim, molgramostin, GMCSF, CSF, GM-CSF |
| **CTLA4** | Cytotoxic T-Lymphocyte-Associated Protein 4 | ALPS5, CD, GSE, GRD4, CELIAC3, IDDM12, CD152, CTLA-4 |
| **CTSD** | Cathepsin D | ceroid-lipofuscinosis, HEL-S-130P, CLN10, CPSD |
| **CXCL1** | Chemokine (C-X-C Motif) Ligand 1 (Melanoma Growth Stimulating Activity, Alpha) | GROa, MGSA-a, FSP, GRO1, MGSA, GRO-alpha(1-73), NAP-3, SCYB1, alpha), GRO |
| **CXCL10** | Chemokine (C-X-C Motif) Ligand 10 | C7, IFI10, crg-2, gIP-10, gamma-IP10, mob-1, SCYB10, INP10, IP-10 |
| **CXCL8** | Chemokine (C-X-C Motif) Ligand 8 | NAP1, GCP1, LECT, LUCT, LYNAP, NAF, emoctakin, interleukin-8, IL8, GCP-1, MDNCF, MONAP, NAP-1, IL-8 |
| **CXCR3** | Chemokine (C-X-C Motif) Receptor 3 | CD182, CD183, CMKAR3, IP10-R, Mig-R, MigR, GPR9, CKR-L2, CXC-R3, CXCR-3 |
| **DENND2D** | DENN/MADD Domain Containing 2D | RP5-1180E21.2 |
| **DLC1** | DLC1 Rho GTPase Activating Protein | DLC-1, HP, p122-RhoGAP, ARHGAP7, STARD12, KIAA1723 |
| **DPP4** | Dipeptidyl-Peptidase 4 | DPPIV, ADCP2, CD26, ADABP, ADCP-2, TP103 |
| **E2F1** | E2F Transcription Factor 1 | RBAP1, RBP3, RBBP3, E2F-1, PBR3, RBAP-1, RBBP-3 |
| **EGR1** | Early Growth Response 1 | G0S30, KROX-24, TIS8, ZIF-268, AT225, EGR-1, NGFI-A, ZNF225, KROX24 |
| **ELANE** | Elastase, Neutrophil Expressed | medullasin, GE, HNE, NE, PMN-E, elastase-2, SCN1, ELA2, HLE |
| **ERBB2** | Erb-B2 Receptor Tyrosine Kinase 2 | CD340, HER-2, HER-2/neu, TKR1, herstatin, NGL, HER2, NEU, p185erbB2, MLN19 |
| **F5** | Coagulation Factor V (Proaccelerin, Labile Factor) | FVL, PCCF, RPRGL1, THPH2 |
| **FAIM3** | Fas Apoptotic Inhibitory Molecule 3 | FCMR, TOSO |
| **FAM210B** | Family With Sequence Similarity 210, Member B | 5A3, dJ1167H4.1, C20orf108 |
| **FASLG** | Fas Ligand (TNF Superfamily, Member 6) | CD178, ALPS1B, APT1LG1, TNFSF6, FASL, APTL, CD95-L, CD95L |
| **FCGR2B** | Fc Fragment Of IgG, Low Affinity IIb, Receptor (CD32) | CDW32, CD32, FCG2, IGFR2, CD32B, fc-gamma-RIIb, fcRII-b, FCGR2 |
| **FOS** | FBJ Murine Osteosarcoma Viral Oncogene Homolog | p55, AP-1, C-FOS, G0S7 |
| **FOXP3** | Forkhead Box P3 | DIETER, FOXP3delta7, JM2, scurfin, AIID, PIDX, XPID, IPEX |
| **FYN** | FYN Proto-Oncogene, Src Family Tyrosine Kinase | SYN, p59-FYN, SLK |
| **GADD45A** | Growth Arrest And DNA-Damage-Inducible, Alpha | DDIT1, GADD45, DDIT-1 |
| **GLRX5** | Glutaredoxin 5 | GRX5, PR01238, PRSA, FLB4739, PRO1238, C14orf87 |
| **GYPA** | Glycophorin A (MNS Blood Group) | MN, CD235a, GPErik, GPSAT, HGpMiV, HGpMiXI, HGpSta(C), glycophorin-A, MNS, GPA, PAS-2 |
| **GYPB** | Glycophorin B (MNS Blood Group) | MNS, CD235b, glycophorin-B, SS, GPB, PAS-3 |
| **GZMA** | Granzyme A (Granzyme 1, Cytotoxic T-Lymphocyte-Associated Serine Esterase 3) | fragmentin-1, CTLA3, HFSP, HF, Granzyme-1 |
| **GZMB** | Granzyme B (Granzyme 2, Cytotoxic T-Lymphocyte-Associated Serine Esterase 1) | HLP, CTLA1, CCPI, CGL-1, CSP-B, fragmentin-2, CSPB, C11, CGL1, CTLA-1, CTSGL1, SECT, GRB, Granzyme-2 |
| **HLA-DRA** | Major Histocompatibility Complex, Class II, DR Alpha | MLRW, HLA-DRA1 |
| **HMGA1** | High Mobility Group AT-Hook 1 | HMG-R, HMGA1A, HMGIY, HMG-I(Y) |
| **HMGB1** | High Mobility Group Box 1 | Amphoterin, HMG3, SBP-1, HMG1, HMG-1 |
| **HMOX1** | Heme Oxygenase 1 | HSP32, bK286B10, HMOX1D, HO-1, HO, HO1 |
| **HOXA10** | Homeobox A10 | PL, HOX1.8, HOX1, HOX1H |
| **HSPA1A** | Heat Shock 70kDa Protein 1A | HEL-S-103, HSP70-1, HSP70-1A, HSP70I, HSP72, HSPA1, HSP70-1/HSP70-2, HSP70.1/HSP70.2, HSX70 |
| **ICAM1** | Intercellular Adhesion Molecule 1 | BB2, CD54, P3.58, ICAM-1 |
| **ICOS** | Inducible T-Cell Co-Stimulator | CD278, CVID1, AILIM |
| **IFI16** | Interferon, Gamma-Inducible Protein 16 | PYHIN2, IFNGIP1, Ifi-16 |
| **IFNG** | Interferon, Gamma | IFG, IFI, IFN-gamma |
| **IGF2BP2** | Insulin-Like Growth Factor 2 MRNA Binding Protein 2 | IMP2, IMP-2, VICKZ2 |
| **IGHG2** | Immunoglobulin Heavy Constant Gamma 2 (G2m Marker) |  |
| **IL10** | Interleukin 10 | IL10A, TGIF, interleukin-10, GVHDS, CSIF, IL-10 |
| **IL12B** | Interleukin 12B | p40, IL12, CLMF, CLMF2, IMD28, IMD29, NKSF, NKSF2, IL-12B |
| **IL15** | Interleukin 15 | interleukin-15, IL-15 |
| **IL18** | Interleukin 18 | IL-1g, iboctadekin, interleukin-18, IGIF, IL-18, IL1F4 |
| **IL18BP** | Interleukin 18 Binding Protein | IL18BPa, tadekinig-alfa, IL-18BP |
| **IL1B** | Interleukin 1, Beta | IL-1, IL1-BETA, catabolin, pro-interleukin-1-beta, IL1F2 |
| **IL1R1** | Interleukin 1 Receptor, Type I | p80, IL1RA, CD121A, D2S1473, IL1R, IL-1R-1, IL-1R-alpha, IL-1RT-1, IL-1RT1, IL1RT1 |
| **IL1R2** | Interleukin 1 Receptor, Type II | CD121b, IL1R2c, IL1RB, CDw121b, IL-1R-2, IL-1R-beta, IL-1RT-2, IL-1RT2 |
| **IL1RN** | Interleukin 1 Receptor Antagonist | IL-1ra3, DIRA, MVCD4, ICIL-1RA, IL-1RN, IL-1ra, IL1F3, IL1RA, IRAP, Anakinra |
| **IL2** | Interleukin 2 | aldesleukin, interleukin-2, lymphokine, IL-2, TCGF |
| **IL23A** | Interleukin 23, Alpha Subunit P19 | p19, interleukin-six, IL-23, IL-23A, IL23P19, SGRF, IL-23-A, IL-23p19 |
| **IL2RA** | Interleukin 2 Receptor, Alpha | p55, CD25, TCGFR, IDDM10, IL2R, IL-2-RA, IL2-RA |
| **IL32** | Interleukin 32 | IL-32alpha, IL-32beta, IL-32delta, IL-32gamma, TAIFa, TAIFb, TAIFc, TAIFd, interleukin-32, NK4, TAIF, IL-32 |
| **IL5** | Interleukin 5 | TRF, interleukin-5, eosinophil, EDF, IL-5 |
| **IL6** | Interleukin 6 | interferon, interleukin-6, BSF2, HGF, HSF, IFNB2, BSF-2, CDF, IFN-beta-2, IL-6 |
| **IL7R** | Interleukin 7 Receptor | CDW127, ILRA, CD127, IL7RA, IL-7R-alpha, IL-7RA |
| **INPP4B** | Inositol Polyphosphate-4-Phosphatase, Type II, 105kDa |  |
| **IRAK3** | Interleukin-1 Receptor-Associated Kinase 3 | ASRT5, IRAKM, IRAK-3, IRAK-M |
| **IRF1** | Interferon Regulatory Factor 1 | MAR, IRF-1 |
| **ITGA4** | Integrin, Alpha 4 (Antigen CD49D, Alpha 4 Subunit Of VLA-4 Receptor) | IA4, CD49D |
| **ITGAL** | Integrin, Alpha L (Antigen CD11A (P180), Lymphocyte Function-Associated Antigen 1; Alpha Polypeptide) | LFA-1, LFA1A, CD11A, LFA-1A |
| **LARGE** | Like-Glycosyltransferase | like-glycosyltransferase, MDC1D, MDDGA6, MDDGB6, KIAA0609, LARGE1 |
| **LCK** | LCK Proto-Oncogene, Src Family Tyrosine Kinase | Lsk, IMD22, YT16, p56lck, pp58lck, p56-LCK |
| **LGALS3** | Lectin, Galactoside-Binding, Soluble, 3 | L31, CBP35, GAL3, GALIG, galectin-3, LGALS2, GALBP, MAC2, Gal-3, L-31 |
| **LTA** | Lymphotoxin Alpha | LT, lymphotoxin-alpha, TNFB, LT-alpha, TNF-beta, TNFSF1 |
| **MAPK14** | Mitogen-Activated Protein Kinase 14 | P38, RK, CSBP, EXIP, Mxi2, PRKM14, PRKM15, p38ALPHA, CSBP2, CSPB1, CSBP1, SAPK2A |
| **MCAM** | Melanoma Cell Adhesion Molecule | Gicerin, CD146, MUC18 |
| **MIF** | Macrophage Migration Inhibitory Factor (Glycosylation-Inhibiting Factor) | GLIF, GIF, MMIF |
| **MMP12** | Matrix Metallopeptidase 12 | HME, ME, MME, MMP-12 |
| **MMP9** | Matrix Metallopeptidase 9 | MANDP2, CLG4B, GELB, MMP-9 |
| **MNDA** | Myeloid Cell Nuclear Differentiation Antigen | PYHIN3 |
| **MSH2** | MutS Homolog 2 | HNPCC, LCFS2, FCC1, HNPCC1, COCA1, hMSH2 |
| **MYC** | V-Myc Avian Myelocytomatosis Viral Oncogene Homolog | MRTL, MYCC, c-Myc, bHLHe39 |
| **NAB2** | NGFI-A Binding Protein 2 (EGR1 Binding Protein 2) | MADER |
| **NBEA** | Neurobeachin | LYST2, neurobeachin, BCL8B, KIAA1544 |
| **NEDD4L** | Neural Precursor Cell Expressed, Developmentally Down-Regulated 4-Like, E3 Ubiquitin Protein Ligase | NEDD4-2, hNEDD4-2, RSP5, NEDD4.2, KIAA0439, NEDL3 |
| **NEDD9** | Neural Precursor Cell Expressed, Developmentally Down-Regulated 9 | P105, Cas-like, CAS2, HEF1, CASL, CAS-L, CASS2, NEDD-9 |
| **NFATC1** | Nuclear Factor Of Activated T-Cells, Cytoplasmic, Calcineurin-Dependent 1 | NF-ATC, NFAT2, NFATc, NF-ATc1, NFATc1 |
| **NFKB1** | Nuclear Factor Of Kappa Light Polypeptide Gene Enhancer In B-Cells 1 | P50, P105, KBF1, NF-kB1, NF-kappa-B, NF-kappaB, NF-kappabeta, NFKB-p105, NFKB-p50, NFkappaB, EBP-1 |
| **NLRC4** | NLR Family, CARD Domain Containing 4 | AIFEC, CLANA, CLANB, CLANC, CLAND, CLR2.1, FCAS4, CARD12, CLAN, IPAF, CLAN1 |
| **NME4** | NME/NM23 Nucleoside Diphosphate Kinase 4 | NDK, NDPK-D, NM23H4, NDPKD, nm23-H4, NM23D |
| **NRAS** | Neuroblastoma RAS Viral (V-Ras) Oncogene Homolog | HRAS1, CMNS, N-ras, NCMS, NRAS1, ALPS4, NS6 |
| **NUCKS1** | Nuclear Casein Kinase And Cyclin-Dependent Kinase Substrate 1 | P1, JC7, NUCKS |
| **NUDT4** | Nudix (Nucleoside Diphosphate Linked Moiety X)-Type Motif 4 | DIPP2alpha, DIPP2beta, HDCMB47P, DIPP-2, DIPP2, KIAA0487 |
| **PBX1** | Pre-B-Cell Leukemia Homeobox 1 | PRL |
| **PDE3B** | Phosphodiesterase 3B, CGMP-Inhibited | HcGIP1, cGIPDE1, CGIP1 |
| **PDGFA** | Platelet-Derived Growth Factor Alpha Polypeptide | PDGF-A, PDGF-1, PDGF1 |
| **PLA2G7** | Phospholipase A2, Group VII (Platelet-Activating Factor Acetylhydrolase, Plasma) | LDL-PLA2, LP-PLA2, PAFAD, PAFAH, LDL-PLA(2), gVIIA-PLA2 |
| **PLAUR** | Plasminogen Activator, Urokinase Receptor | CD87, URKR, U-PAR, UPAR, MO3 |
| **PLEK2** | Pleckstrin 2 | pleckstrin-2 |
| **PLXDC2** | Plexin Domain Containing 2 | 1200007L24Rik, TEM7R |
| **PPP2R4** | Protein Phosphatase 2A Activator, Regulatory Subunit 4 | PTPA, PP2A, PR53 |
| **PTEN** | Phosphatase And Tensin Homolog | 10q23del, DEC, PTEN1, BZS, MHAM, CWS1, GLM2, MMAC1, TEP1 |
| **PTGS2** | Prostaglandin-Endoperoxide Synthase 2 (Prostaglandin G/H Synthase And Cyclooxygenase) | COX2, PGG/HS, GRIPGHS, PHS-2, hCox-2, COX-2, PGHS-2, Cyclooxygenase-2 |
| **PTPRC** | Protein Tyrosine Phosphatase, Receptor Type, C | LCA, GP180, B220, CD45R, LY5, CD45, L-CA, T200 |
| **PTPRK** | Protein Tyrosine Phosphatase, Receptor Type, K | R-PTP-kappa, PTPK |
| **RBM5** | RNA Binding Motif Protein 5 | G15, H37, LUCA15, RMB5 |
| **RHOC** | Ras Homolog Family Member C | H9, RHOH9, ARH9, ARHC |
| **S100A4** | S100 Calcium Binding Protein A4 | MTS1, 18A2, 42A, FSP1, P9KA, PEL98, CAPL, Calvasculin, Metastasin |
| **S100A6** | S100 Calcium Binding Protein A6 | 2A9, 5B10, CABP, calcyclin, CACY, PRA |
| **SCN3A** | Sodium Channel, Voltage Gated, Type III Alpha Subunit | Nav1.3, NAC3, KIAA1356 |
| **SERPINA1** | Serpin Peptidase Inhibitor, Clade A (Alpha-1 Antiproteinase, Antitrypsin), Member 1 | PI, alpha-1-antitrypsin, A1A, A1AT, PI1, PRO2275, alpha-1-antiproteinase, alpha1AT, AAT |
| **SERPINE1** | Serpin Peptidase Inhibitor, Clade E (Nexin, Plasminogen Activator Inhibitor Type 1), Member 1 | PAI, PAI1, PLANH1, PAI-1, PLANH1 |
| **SIAH2** | Siah E3 Ubiquitin Protein Ligase 2 | siah-2, hSiah2 |
| **SLC4A1** | Solute Carrier Family 4 (Anion Exchanger), Member 1 (Diego Blood Group) | BND3, CD233, EMPB3, FR, RTA1A, SW, WD1, WR, WD, DI, AE1, EPB3 |
| **SOCS1** | Suppressor Of Cytokine Signaling 1 | CIS1, CISH1, SSI1, JAB, SOCS-1, SSI-1, TIP-3, TIP3 |
| **SOCS3** | Suppressor Of Cytokine Signaling 3 | ATOD4, Cish3, CIS3, SSI3, SOCS-3, SSI-3, CIS-3 |
| **SPARC** | Secreted Protein, Acidic, Cysteine-Rich (Osteonectin) | osteonectin, ON, BM-40 |
| **ST14** | Suppression Of Tumorigenicity 14 (Colon Carcinoma) | epithin, matriptase, HAI, TMPRSS14, prostamin, PRSS14, ARCI11, MTSP1, MT-SP1, SNC19, TADG15 |
| **TGFB1** | Transforming Growth Factor, Beta 1 | LAP, CED, TGFbeta, TGFB, DPD1, TGF-beta-1 |
| **THBS1** | Thrombospondin 1 | TSP1, thrombospondin-1p180, THBS, THBS-1, TSP-1, thrombospondin-1, TSP |
| **TIMP1** | TIMP Metallopeptidase Inhibitor 1 | EPO, HCI, CLGI, TIMP, EPA, TIMP-1 |
| **TLK2** | Tousled-Like Kinase 2 | PKU-ALPHA, HsHPK |
| **TLR2** | Toll-Like Receptor 2 | CD282, TIL4 |
| **TLR4** | Toll-Like Receptor 4 | CD284, TLR-4, TOLL, ARMD10, hToll |
| **TLR9** | Toll-Like Receptor 9 | CD289 |
| **TMOD1** |  | ETMOD, e-tropomodulin, tropomodulin-1, D9S57E, TMOD, E-Tmod |
| **TNF** | Tumor Necrosis Factor | DIF, cachectin, TNFA, TNF-a, TNF-alpha, TNFSF2 |
| **TNFRSF13B** | Tumor Necrosis Factor Receptor Superfamily, Member 13B | CD267, CVID, IGAD2, RYZN, TNFRSF14B, CVID2, TACI |
| **TNFRSF1A** | Tumor Necrosis Factor Receptor Superfamily, Member 1A | P60, tbp1, p55, CD120a, TNF-R, TNF-R-I, TNF-R55, TNFR1-d2, TNFR55, TNFR60, p55-R, FPF, MS5, TNFR1, TNFAR, TNF-R1, TNF-RI, TNFR-I |
| **TNFRSF1B** | Tumor Necrosis Factor Receptor Superfamily, Member 1B | p75, CD120b, TBPII, TNF-R-II, TNF-R75, TNFR1B, TNFR80, p75TNFR, TNFR2, TNFBR, TNF-R2, TNF-RII, Etanercept, TNFR-II |
| **TNS1** | Tensin 1 | tensin, MST091, MST122, MST127, MSTP091, MSTP122, MSTP127, PPP1R155, tensin-1, MXRA6, TNS |
| **TP53** | Tumor Protein P53 | TRP53, BCC7, LFS1, P53 |
| **TSPAN5** | Tetraspanin 5 | NET-4, TSPAN-5, tetraspanin-5, TM4SF9, NET4 |
| **TXNRD1** | Thioredoxin Reductase 1 | TR1, TR, TRXR1, oxidoreductase, TXNR, GRIM-12, GRIM12, KDRF |
| **UBE2C** | Ubiquitin-Conjugating Enzyme E2C | dJ447F3.2, UBCH10 |
| **VEGFA** | Vascular Endothelial Growth Factor A | MVCD1, VEGF, VPF, VEGF-A |
| **XK** | X-Linked Kx Blood Group | NAC, neuroacanthocytosis, neurocanthocytosis, KX, X1k, NA, MCLDS, XKR1, XRG1 |
