# Supplementary material for: A whole-blood RNA transcript-based gene signature is associated with the development of CTLA-4 blockade-related diarrhea in patients with advanced melanoma treated with the checkpoint inhibitor tremelimumab
Source: J Immunother Cancer. 2018 Sep 18;6:90. doi: 10.1186/s40425-018-0408-9 (PMC6145108; doi:10.1186/s40425-018-0408-9)
Supplement: Supplementary file 2 — Table S2 Differences in pre-treatment and post-treatment expression of each of the 169 genes tested between patients who developed grade 0–1 versus grade 2–4 diarrhea. (a) discovery dataset and (b) validation dataset. (DOCX 17 kb) [file 40425_2018_408_MOESM2_ESM.docx]

a.

|  |  |  |  |  |
| --- | --- | --- | --- | --- |
| Worst Diarrhea Grade | Phase II Discovery Dataset Actual N=150 | 16 Gene Signature Diarrhea Prediction | Patients Incorrectly Classified | 16 Gene Signature % Correct Classification |
|  |  |  |  |  |
| Grade 4 | 0 | 0 | 0 | 0 |
| Grade 3 | 9 | 7 | 2 | 77.8% |
| Grade 2 | 12 | 7 | 5 | 58.3% |
| subtotal | 21 | 14 | 7 | 66.7% |
| Grade 1 | 39 | 10 | 10 | 74.4% |
| Grade 0 | 90 | 23 | 23 | 74.4% |
| subtotal | 129 | 33 | 33 | 74.4% |
| Total | 150 | 47 | 40 | 73.3% |

b.

|  |  |  |  |  |
| --- | --- | --- | --- | --- |
| Worst Diarrhea Grade | Phase III Validation Dataset Actual N=210 | 16 Gene Signature Diarrhea Prediction | Patients Incorrectly Classified | 16 Gene Signature % Correct Classification |
|  |  |  |  |  |
| Grade 4 | 1 | 1 | 0 | 100.0% |
| Grade 3 | 26 | 25 | 1 | 96.2% |
| Grade 2 | 29 | 24 | 5 | 82.8% |
| subtotal | 56 | 50 | 6 | 89.3% |
| Grade 1 | 36 | 24 | 24 | 33.3% |
| Grade 0 | 118 | 50 | 50 | 57.6% |
| subtotal | 154 | 74 | 74 | 51.9% |
| Total | 210 | 124 | 80 | 61.9% |
