# Supplementary material for: A whole-blood RNA transcript-based gene signature is associated with the development of CTLA-4 blockade-related diarrhea in patients with advanced melanoma treated with the checkpoint inhibitor tremelimumab
Source: J Immunother Cancer. 2018 Sep 18;6:90. doi: 10.1186/s40425-018-0408-9 (PMC6145108; doi:10.1186/s40425-018-0408-9)
Supplement: Supplementary file 3 — Table S3 (a) Using a − 1.70 cut off when testing the 16-gene signature on the discovery data set showed a 66.7% sensitivity, 74.4% specificity, 29.8% PPV, and 93.2% NPV with correct classification of 73.3% of patients. Accuracy of patient classification of as a function of diarrhea grade using the 16-gene signature. (b) Applying this − 1.70 cut off to the validation data set revealed an 89.3% sensitivity, 51.9% specificity, 40.3% PPV, and 93% NPV with correct classification of 61.9% of patients. Accuracy of patient classification of as a function of diarrhea grade using the 16-gene signature. (DOCX 92 kb) [file 40425_2018_408_MOESM3_ESM.docx]

|  | |  |  |  |  |  |  |  |  |  |  |  |  |  |  |  |
| --- | --- | --- | --- | --- | --- | --- | --- | --- | --- | --- | --- | --- | --- | --- | --- | --- |
|  | | | | | | |  |  |  |  |  |  |  |  |  |  |
|  |  |  |  |  |  |  |  |  |  |  |  |  |  |  |  |  |
| **Predictor Gene Name** | **Gene Expression Delta Ct Values Discovery Dataset (N=150)** | | | | | | |  | **Predictor Gene Name** | **Gene Expression Delta Ct Values Validation Dataset (N=210)** | | | | | | |
|  | **Grade 0-1 Diarrhea Pre Treatment N=129** | **Grade 0-1 Diarrhea Post Treatment N=129** | **Grade 0-1 Diarrhea Pre Post Difference** | **Grade 2-4 Diarrhea Pre Treatment N=21** | **Grade 2-4 Diarrhea Post Treatment N=21** | **Grade 2-4 Diarrhea Pre Post Difference** | **Grade 2-4 less Grade 0-1 Diarrhea Pre Post Difference** |  |  | **Grade 0-1 Diarrhea Pre Treatment N=154** | **Grade 0-1 Diarrhea Post Treatment N=154** | **Grade 0-1 Diarrhea Pre Post Difference** | **Grade 2-4 Diarrhea Pre Treatment N=56** | **Grade 2-4 Diarrhea Post Treatment N=56** | **Grade 2-4 Diarrhea Pre Post Difference** | **Grade 2-4 less Grade 0-1 Diarrhea Pre Post Difference** |
| **ADAM17** | **18.29** | **18.32** | **0.04** | **18.32** | **18.24** | **-0.08** | **-0.12** |  | **ADAM17** | **18.32** | **18.25** | **-0.07** | **18.38** | **18.31** | **-0.07** | **0.00** |
| **ALOX5** | **15.96** | **15.98** | **0.03** | **16.08** | **15.67** | **-0.41** | **-0.44** |  | **ALOX5** | **16.37** | **16.25** | **-0.12** | **16.54** | **16.06** | **-0.49** | **-0.37** |
| **ANLN** | **22.20** | **21.59** | **-0.60** | **22.26** | **21.19** | **-1.07** | **-0.47** |  | **ANLN** | **22.32** | **21.37** | **-0.96** | **22.38** | **21.22** | **-1.16** | **-0.21** |
| **APAF1** | **17.16** | **17.33** | **0.16** | **17.13** | **17.26** | **0.12** | **-0.04** |  | **APAF1** | **17.17** | **17.24** | **0.07** | **17.21** | **17.20** | **-0.01** | **-0.08** |
| **AXIN2** | **20.35** | **20.09** | **-0.26** | **19.89** | **19.95** | **0.06** | **0.32** |  | **AXIN2** | **19.78** | **19.61** | **-0.17** | **19.79** | **19.69** | **-0.10** | **0.07** |
| **BAD** | **18.64** | **18.55** | **-0.09** | **18.60** | **18.43** | **-0.17** | **-0.08** |  | **BAD** | **18.63** | **18.43** | **-0.20** | **18.66** | **18.43** | **-0.23** | **-0.04** |
| **BAX** | **16.09** | **16.03** | **-0.07** | **16.10** | **16.00** | **-0.10** | **-0.03** |  | **BAX** | **15.77** | **15.67** | **-0.10** | **15.80** | **15.66** | **-0.15** | **-0.05** |
| **BLVRB** | **13.51** | **13.69** | **0.18** | **13.56** | **13.56** | **0.00** | **-0.18** |  | **BLVRB** | **13.87** | **13.88** | **0.02** | **14.01** | **13.90** | **-0.11** | **-0.13** |
| **BPGM** | **16.40** | **16.68** | **0.28** | **16.44** | **16.57** | **0.14** | **-0.14** |  | **BPGM** | **17.21** | **17.13** | **-0.08** | **17.55** | **17.39** | **-0.16** | **-0.08** |
| **BRCA1** | **21.57** | **21.60** | **0.03** | **21.66** | **21.60** | **-0.06** | **-0.09** |  | **BRCA1** | **21.65** | **21.49** | **-0.15** | **21.66** | **21.47** | **-0.19** | **-0.04** |
| **C1QA** | **19.96** | **19.61** | **-0.35** | **20.42** | **19.39** | **-1.02** | **-0.67** |  | **C1QA** | **20.00** | **19.64** | **-0.36** | **20.30** | **19.31** | **-0.99** | **-0.62** |
| **CARD12** | **17.63** | **17.67** | **0.04** | **17.73** | **17.25** | **-0.47** | **-0.52** |  | **CARD12** | **17.77** | **17.68** | **-0.09** | **17.95** | **17.34** | **-0.61** | **-0.53** |
| **CASP1** | **15.64** | **15.61** | **-0.03** | **15.74** | **15.50** | **-0.24** | **-0.21** |  | **CASP1** | **15.62** | **15.50** | **-0.13** | **15.76** | **15.50** | **-0.26** | **-0.13** |
| **CASP3** | **19.90** | **19.58** | **-0.32** | **19.87** | **19.56** | **-0.31** | **0.01** |  | **CASP3** | **20.38** | **19.97** | **-0.41** | **20.51** | **20.03** | **-0.48** | **-0.06** |
| **CCL3** | **20.60** | **20.63** | **0.03** | **20.90** | **21.00** | **0.10** | **0.07** |  | **CCL3** | **20.83** | **20.92** | **0.08** | **21.09** | **21.23** | **0.14** | **0.06** |
| **CCL5** | **12.95** | **12.82** | **-0.13** | **13.11** | **12.80** | **-0.31** | **-0.18** |  | **CCL5** | **13.19** | **12.96** | **-0.23** | **13.33** | **13.05** | **-0.28** | **-0.05** |
| **CCND1** | **23.69** | **23.89** | **0.21** | **23.63** | **24.24** | **0.61** | **0.40** |  | **CCND1** | **23.21** | **23.43** | **0.22** | **23.40** | **23.62** | **0.22** | **0.00** |
| **CCR3** | **16.71** | **16.41** | **-0.30** | **16.98** | **17.40** | **0.42** | **0.72** |  | **CCR3** | **16.78** | **16.57** | **-0.21** | **16.99** | **17.41** | **0.42** | **0.63** |
| **CCR5** | **18.06** | **17.59** | **-0.47** | **17.93** | **17.44** | **-0.49** | **-0.03** |  | **CCR5** | **17.93** | **17.31** | **-0.62** | **18.08** | **17.47** | **-0.61** | **0.01** |
| **CCR7** | **16.07** | **15.82** | **-0.25** | **15.65** | **15.56** | **-0.09** | **0.16** |  | **CCR7** | **15.70** | **15.54** | **-0.16** | **15.65** | **15.50** | **-0.15** | **0.01** |
| **CCR9** | **23.59** | **22.57** | **-1.02** | **23.31** | **22.48** | **-0.83** | **0.19** |  | **CCR9** | **23.66** | **22.72** | **-0.94** | **23.75** | **22.70** | **-1.04** | **-0.10** |
| **CD19** | **20.17** | **20.06** | **-0.11** | **19.98** | **20.23** | **0.25** | **0.37** |  | **CD19** | **19.56** | **19.65** | **0.10** | **19.61** | **19.59** | **-0.01** | **-0.11** |
| **CD28** | **17.82** | **16.99** | **-0.83** | **17.59** | **16.88** | **-0.71** | **0.13** |  | **CD28** | **17.65** | **16.83** | **-0.82** | **17.67** | **16.90** | **-0.77** | **0.04** |
| **CD4** | **15.25** | **15.11** | **-0.15** | **15.21** | **15.24** | **0.02** | **0.17** |  | **CD4** | **15.36** | **15.16** | **-0.20** | **15.43** | **15.27** | **-0.15** | **0.04** |
| **CD40** | **19.99** | **19.95** | **-0.04** | **19.98** | **19.78** | **-0.20** | **-0.16** |  | **CD40** | **19.92** | **19.86** | **-0.06** | **19.96** | **19.83** | **-0.14** | **-0.07** |
| **CD80** | **24.39** | **24.20** | **-0.19** | **24.24** | **24.09** | **-0.15** | **0.04** |  | **CD80** | **24.36** | **24.04** | **-0.32** | **24.27** | **24.13** | **-0.14** | **0.18** |
| **CD86** | **17.83** | **17.90** | **0.07** | **17.94** | **17.83** | **-0.12** | **-0.19** |  | **CD86** | **17.97** | **17.91** | **-0.06** | **18.07** | **17.88** | **-0.20** | **-0.14** |
| **CD8A** | **16.41** | **16.07** | **-0.34** | **16.36** | **16.07** | **-0.29** | **0.06** |  | **CD8A** | **16.13** | **15.76** | **-0.37** | **16.17** | **15.94** | **-0.23** | **0.14** |
| **CD97** | **13.11** | **13.07** | **-0.04** | **13.19** | **13.20** | **0.01** | **0.04** |  | **CD97** | **12.97** | **12.96** | **0.00** | **13.00** | **13.08** | **0.08** | **0.09** |
| **CDC25A** | **23.40** | **22.10** | **-1.30** | **23.67** | **21.93** | **-1.74** | **-0.44** |  | **CDC25A** | **23.45** | **21.84** | **-1.61** | **23.64** | **21.45** | **-2.19** | **-0.58** |
| **CDH1** | **20.23** | **20.44** | **0.20** | **20.38** | **20.56** | **0.18** | **-0.02** |  | **CDH1** | **20.94** | **20.90** | **-0.04** | **21.10** | **20.92** | **-0.18** | **-0.14** |
| **CDK2** | **19.96** | **19.71** | **-0.25** | **19.86** | **19.61** | **-0.26** | **-0.01** |  | **CDK2** | **19.87** | **19.55** | **-0.32** | **19.93** | **19.51** | **-0.42** | **-0.10** |
| **CDKN1A** | **16.97** | **17.14** | **0.17** | **17.18** | **17.22** | **0.05** | **-0.12** |  | **CDKN1A** | **17.08** | **17.12** | **0.04** | **17.11** | **17.03** | **-0.09** | **-0.13** |
| **CDKN1B** | **15.53** | **15.43** | **-0.09** | **15.44** | **15.32** | **-0.12** | **-0.03** |  | **CDKN1B** | **15.11** | **15.00** | **-0.11** | **15.13** | **15.02** | **-0.11** | **0.00** |
| **CDKN2A** | **21.28** | **20.49** | **-0.79** | **21.22** | **20.41** | **-0.81** | **-0.02** |  | **CDKN2A** | **21.08** | **20.20** | **-0.88** | **21.18** | **20.13** | **-1.06** | **-0.18** |
| **CDKN2D** | **15.11** | **15.24** | **0.12** | **15.24** | **15.13** | **-0.11** | **-0.23** |  | **CDKN2D** | **15.02** | **15.02** | **-0.01** | **15.06** | **14.92** | **-0.14** | **-0.13** |
| **CHPT1** | **16.45** | **16.60** | **0.15** | **16.54** | **16.39** | **-0.15** | **-0.30** |  | **CHPT1** | **16.81** | **16.71** | **-0.10** | **16.93** | **16.72** | **-0.21** | **-0.10** |
| **CNKSR2** | **22.35** | **22.06** | **-0.29** | **22.02** | **22.00** | **-0.02** | **0.26** |  | **CNKSR2** | **22.10** | **21.88** | **-0.23** | **22.08** | **21.98** | **-0.10** | **0.13** |
| **CSF2** | **25.02** | **24.95** | **-0.07** | **25.12** | **25.03** | **-0.09** | **-0.01** |  | **CSF2** | **25.32** | **25.07** | **-0.25** | **25.33** | **25.09** | **-0.24** | **0.02** |
| **CTLA4** | **19.97** | **18.56** | **-1.41** | **19.63** | **18.50** | **-1.13** | **0.28** |  | **CTLA4** | **19.75** | **18.21** | **-1.55** | **19.68** | **18.21** | **-1.47** | **0.08** |
| **CTLA4SOL** | **25.42** | **25.41** | **-0.01** | **25.42** | **25.33** | **-0.10** | **-0.09** |  | **CTLA4SOL** | **25.37** | **25.29** | **-0.08** | **25.29** | **25.32** | **0.02** | **0.11** |
| **CTSD** | **13.23** | **13.27** | **0.05** | **13.38** | **13.09** | **-0.29** | **-0.33** |  | **CTSD** | **13.10** | **12.98** | **-0.12** | **13.19** | **12.92** | **-0.26** | **-0.14** |
| **CXCL1** | **19.80** | **20.10** | **0.30** | **19.87** | **20.09** | **0.22** | **-0.08** |  | **CXCL1** | **20.20** | **20.52** | **0.32** | **20.31** | **20.60** | **0.29** | **-0.03** |
| **CXCL10** | **23.58** | **23.37** | **-0.21** | **23.84** | **23.48** | **-0.36** | **-0.16** |  | **CXCL10** | **24.24** | **23.85** | **-0.39** | **24.71** | **23.90** | **-0.81** | **-0.41** |
| **CXCR3** | **18.20** | **17.33** | **-0.87** | **18.03** | **17.25** | **-0.78** | **0.09** |  | **CXCR3** | **17.97** | **17.08** | **-0.89** | **18.06** | **17.20** | **-0.86** | **0.03** |
| **DLC1** | **23.54** | **23.74** | **0.20** | **23.68** | **23.73** | **0.05** | **-0.15** |  | **DLC1** | **23.92** | **23.91** | **-0.02** | **24.16** | **23.65** | **-0.51** | **-0.50** |
| **DPP4** | **18.93** | **18.82** | **-0.11** | **18.72** | **18.76** | **0.03** | **0.14** |  | **DPP4** | **18.91** | **18.80** | **-0.11** | **18.88** | **18.80** | **-0.08** | **0.03** |
| **E2F1** | **20.98** | **20.46** | **-0.52** | **21.07** | **20.27** | **-0.80** | **-0.28** |  | **E2F1** | **21.00** | **20.40** | **-0.60** | **21.28** | **20.21** | **-1.07** | **-0.47** |
| **EGR1** | **19.97** | **20.14** | **0.17** | **20.06** | **20.23** | **0.17** | **0.00** |  | **EGR1** | **20.24** | **20.14** | **-0.10** | **20.24** | **20.04** | **-0.21** | **-0.11** |
| **ELA2** | **20.21** | **20.44** | **0.24** | **20.14** | **19.59** | **-0.55** | **-0.79** |  | **ELA2** | **20.17** | **19.83** | **-0.34** | **20.07** | **19.40** | **-0.66** | **-0.32** |
| **ERBB2** | **23.34** | **23.27** | **-0.07** | **23.24** | **23.40** | **0.16** | **0.23** |  | **ERBB2** | **23.13** | **22.91** | **-0.22** | **23.31** | **22.93** | **-0.38** | **-0.16** |
| **F5** | **17.98** | **17.83** | **-0.15** | **18.04** | **17.40** | **-0.64** | **-0.49** |  | **F5** | **18.08** | **17.80** | **-0.28** | **18.24** | **17.54** | **-0.70** | **-0.41** |
| **FAM210B** | **15.21** | **15.47** | **0.27** | **15.32** | **15.58** | **0.26** | **0.00** |  | **FAM210B** | **15.86** | **15.89** | **0.03** | **16.24** | **16.29** | **0.05** | **0.02** |
| **FCGR2B** | **12.66** | **12.67** | **0.01** | **12.80** | **12.61** | **-0.19** | **-0.20** |  | **FCGR2B** | **12.38** | **12.45** | **0.07** | **12.51** | **12.43** | **-0.08** | **-0.15** |
| **FOS** | **15.97** | **16.15** | **0.19** | **16.04** | **16.16** | **0.12** | **-0.06** |  | **FOS** | **15.94** | **16.20** | **0.26** | **15.99** | **16.32** | **0.33** | **0.07** |
| **FOXP3** | **20.47** | **19.63** | **-0.84** | **20.23** | **19.55** | **-0.68** | **0.16** |  | **FOXP3** | **20.53** | **19.62** | **-0.91** | **20.49** | **19.66** | **-0.83** | **0.08** |
| **FYN** | **15.57** | **15.37** | **-0.21** | **15.47** | **15.32** | **-0.15** | **0.06** |  | **FYN** | **15.25** | **15.04** | **-0.22** | **15.26** | **15.08** | **-0.19** | **0.03** |
| **GADD45A** | **19.86** | **19.77** | **-0.09** | **20.05** | **19.28** | **-0.76** | **-0.68** |  | **GADD45A** | **19.97** | **19.66** | **-0.30** | **20.06** | **19.22** | **-0.84** | **-0.54** |
| **GLRX5** | **14.87** | **15.05** | **0.18** | **14.99** | **15.01** | **0.02** | **-0.16** |  | **GLRX5** | **15.37** | **15.30** | **-0.07** | **15.60** | **15.31** | **-0.29** | **-0.22** |
| **GYPA** | **19.40** | **19.59** | **0.19** | **19.29** | **19.29** | **0.00** | **-0.19** |  | **GYPA** | **20.27** | **20.19** | **-0.08** | **20.58** | **20.48** | **-0.10** | **-0.02** |
| **GYPB** | **18.10** | **18.38** | **0.29** | **18.28** | **18.36** | **0.07** | **-0.21** |  | **GYPB** | **19.12** | **19.03** | **-0.08** | **19.52** | **19.40** | **-0.12** | **-0.04** |
| **GZMA** | **17.62** | **17.24** | **-0.38** | **17.67** | **17.22** | **-0.44** | **-0.06** |  | **GZMA** | **17.41** | **17.01** | **-0.39** | **17.50** | **17.14** | **-0.35** | **0.04** |
| **GZMB** | **17.25** | **17.14** | **-0.10** | **17.28** | **17.30** | **0.02** | **0.12** |  | **GZMB** | **17.25** | **17.12** | **-0.12** | **17.28** | **17.24** | **-0.03** | **0.09** |
| **HLADRA** | **12.52** | **12.49** | **-0.02** | **12.73** | **12.48** | **-0.25** | **-0.22** |  | **HLADRA** | **12.59** | **12.50** | **-0.09** | **12.75** | **12.63** | **-0.13** | **-0.04** |
| **HMGA1** | **16.54** | **16.21** | **-0.33** | **16.40** | **16.05** | **-0.35** | **-0.03** |  | **HMGA1** | **16.24** | **15.79** | **-0.44** | **16.23** | **15.74** | **-0.49** | **-0.05** |
| **HMGB1** | **18.32** | **17.96** | **-0.36** | **17.94** | **17.84** | **-0.09** | **0.26** |  | **HMGB1** | **18.17** | **17.89** | **-0.28** | **17.92** | **17.83** | **-0.09** | **0.19** |
| **HMOX1** | **16.40** | **16.52** | **0.12** | **16.60** | **16.46** | **-0.14** | **-0.25** |  | **HMOX1** | **16.26** | **16.36** | **0.10** | **16.42** | **16.34** | **-0.08** | **-0.19** |
| **HOXA10** | **23.21** | **23.25** | **0.05** | **23.72** | **23.18** | **-0.55** | **-0.59** |  | **HOXA10** | **23.02** | **22.97** | **-0.05** | **23.19** | **22.74** | **-0.45** | **-0.40** |
| **HSPA1A** | **14.78** | **14.73** | **-0.05** | **14.84** | **14.57** | **-0.27** | **-0.22** |  | **HSPA1A** | **14.95** | **14.86** | **-0.09** | **15.01** | **14.76** | **-0.25** | **-0.16** |
| **ICAM1** | **17.37** | **17.34** | **-0.04** | **17.41** | **17.19** | **-0.23** | **-0.19** |  | **ICAM1** | **17.50** | **17.37** | **-0.13** | **17.66** | **17.41** | **-0.25** | **-0.12** |
| **ICOS** | **20.02** | **19.12** | **-0.90** | **19.78** | **19.01** | **-0.77** | **0.13** |  | **ICOS** | **19.88** | **18.93** | **-0.95** | **19.79** | **18.96** | **-0.83** | **0.12** |
| **IFI16** | **14.63** | **14.45** | **-0.18** | **14.83** | **14.42** | **-0.41** | **-0.23** |  | **IFI16** | **14.78** | **14.54** | **-0.24** | **14.96** | **14.53** | **-0.43** | **-0.19** |
| **IFNG** | **23.65** | **23.19** | **-0.45** | **23.90** | **23.13** | **-0.76** | **-0.31** |  | **IFNG** | **23.34** | **22.78** | **-0.56** | **23.45** | **22.90** | **-0.55** | **0.01** |
| **IGF2BP2** | **15.98** | **16.15** | **0.17** | **16.15** | **16.20** | **0.05** | **-0.12** |  | **IGF2BP2** | **16.68** | **16.64** | **-0.04** | **16.88** | **16.71** | **-0.17** | **-0.12** |
| **IGHG2** | **19.64** | **18.56** | **-1.08** | **19.05** | **18.00** | **-1.05** | **0.03** |  | **IGHG2** | **18.33** | **17.08** | **-1.25** | **18.65** | **16.77** | **-1.87** | **-0.62** |
| **IL10** | **23.24** | **23.06** | **-0.18** | **23.60** | **22.75** | **-0.85** | **-0.67** |  | **IL10** | **23.37** | **22.90** | **-0.47** | **23.31** | **22.47** | **-0.84** | **-0.36** |
| **IL12B** | **25.41** | **25.61** | **0.20** | **25.40** | **25.37** | **-0.03** | **-0.23** |  | **IL12B** | **25.72** | **25.70** | **-0.01** | **25.70** | **25.75** | **0.05** | **0.06** |
| **IL15** | **21.37** | **21.40** | **0.03** | **21.34** | **21.25** | **-0.09** | **-0.12** |  | **IL15** | **21.44** | **21.32** | **-0.12** | **21.64** | **21.30** | **-0.34** | **-0.22** |
| **IL18** | **21.66** | **21.77** | **0.11** | **21.58** | **21.58** | **0.00** | **-0.12** |  | **IL18** | **21.79** | **21.81** | **0.02** | **21.84** | **21.73** | **-0.10** | **-0.13** |
| **IL18BP** | **17.55** | **17.46** | **-0.09** | **17.42** | **17.46** | **0.04** | **0.13** |  | **IL18BP** | **17.42** | **17.27** | **-0.15** | **17.42** | **17.42** | **0.00** | **0.15** |
| **IL1B** | **16.60** | **16.64** | **0.04** | **16.69** | **16.47** | **-0.21** | **-0.25** |  | **IL1B** | **16.57** | **16.59** | **0.02** | **16.73** | **16.64** | **-0.08** | **-0.11** |
| **IL1R1** | **20.48** | **20.64** | **0.17** | **20.28** | **20.49** | **0.21** | **0.04** |  | **IL1R1** | **20.71** | **20.82** | **0.11** | **20.74** | **20.57** | **-0.17** | **-0.28** |
| **IL1R2** | **16.29** | **16.51** | **0.21** | **16.31** | **16.19** | **-0.12** | **-0.33** |  | **IL1R2** | **16.23** | **16.41** | **0.17** | **16.25** | **16.15** | **-0.11** | **-0.28** |
| **IL1RN** | **16.69** | **16.78** | **0.09** | **16.87** | **16.66** | **-0.22** | **-0.31** |  | **IL1RN** | **16.69** | **16.73** | **0.04** | **16.91** | **16.63** | **-0.28** | **-0.32** |
| **IL2** | **25.40** | **25.45** | **0.05** | **25.31** | **25.32** | **0.01** | **-0.04** |  | **IL2** | **25.43** | **25.39** | **-0.05** | **25.38** | **25.37** | **-0.01** | **0.04** |
| **IL23A** | **21.88** | **21.59** | **-0.30** | **21.54** | **21.37** | **-0.17** | **0.13** |  | **IL23A** | **21.42** | **21.08** | **-0.35** | **21.47** | **21.19** | **-0.28** | **0.07** |
| **IL2RA** | **19.04** | **18.59** | **-0.45** | **18.84** | **18.57** | **-0.27** | **0.18** |  | **IL2RA** | **19.16** | **18.64** | **-0.51** | **19.16** | **18.79** | **-0.37** | **0.15** |
| **IL32** | **14.31** | **13.72** | **-0.59** | **14.22** | **13.76** | **-0.46** | **0.13** |  | **IL32** | **14.26** | **13.67** | **-0.58** | **14.27** | **13.74** | **-0.53** | **0.05** |
| **IL5** | **23.08** | **22.92** | **-0.16** | **23.20** | **23.42** | **0.22** | **0.38** |  | **IL5** | **23.05** | **22.83** | **-0.22** | **23.40** | **23.27** | **-0.13** | **0.09** |
| **IL6** | **25.19** | **25.40** | **0.22** | **25.16** | **25.25** | **0.09** | **-0.13** |  | **IL6** | **25.43** | **25.53** | **0.10** | **25.30** | **25.48** | **0.18** | **0.08** |
| **IL7R** | **15.68** | **15.41** | **-0.27** | **15.31** | **15.10** | **-0.21** | **0.06** |  | **IL7R** | **15.52** | **15.30** | **-0.22** | **15.46** | **15.30** | **-0.16** | **0.06** |
| **IL8** | **22.14** | **22.65** | **0.51** | **21.84** | **23.13** | **1.29** | **0.77** |  | **IL8** | **22.12** | **22.80** | **0.67** | **22.23** | **23.49** | **1.26** | **0.58** |
| **INPP4B** | **18.04** | **17.72** | **-0.32** | **17.81** | **17.64** | **-0.17** | **0.15** |  | **INPP4B** | **17.84** | **17.51** | **-0.33** | **17.51** | **17.59** | **0.08** | **0.40** |
| **IRAK3** | **16.40** | **16.46** | **0.06** | **16.51** | **16.09** | **-0.42** | **-0.48** |  | **IRAK3** | **16.69** | **16.63** | **-0.07** | **16.80** | **16.35** | **-0.45** | **-0.39** |
| **IRF1** | **13.20** | **13.09** | **-0.11** | **13.24** | **13.06** | **-0.18** | **-0.07** |  | **IRF1** | **13.27** | **13.15** | **-0.12** | **13.38** | **13.23** | **-0.16** | **-0.04** |
| **ITGA4** | **14.74** | **14.41** | **-0.33** | **14.71** | **14.48** | **-0.22** | **0.10** |  | **ITGA4** | **14.56** | **14.19** | **-0.37** | **14.28** | **14.30** | **0.03** | **0.39** |
| **ITGAL** | **15.13** | **15.00** | **-0.13** | **15.09** | **15.01** | **-0.08** | **0.05** |  | **ITGAL** | **15.01** | **14.79** | **-0.22** | **15.06** | **14.88** | **-0.18** | **0.04** |
| **LARGE** | **23.82** | **24.03** | **0.20** | **23.64** | **23.82** | **0.18** | **-0.02** |  | **LARGE** | **22.87** | **23.03** | **0.16** | **22.80** | **22.89** | **0.09** | **-0.07** |
| **LCK** | **16.52** | **15.99** | **-0.54** | **16.35** | **15.91** | **-0.44** | **0.10** |  | **LCK** | **16.15** | **15.56** | **-0.59** | **16.12** | **15.62** | **-0.51** | **0.09** |
| **LGALS3** | **17.05** | **17.16** | **0.11** | **17.06** | **17.14** | **0.08** | **-0.03** |  | **LGALS3** | **17.97** | **17.97** | **0.01** | **18.14** | **18.13** | **0.00** | **-0.01** |
| **LTA** | **20.03** | **19.91** | **-0.12** | **19.67** | **19.84** | **0.17** | **0.30** |  | **LTA** | **20.08** | **19.93** | **-0.16** | **20.03** | **19.95** | **-0.08** | **0.08** |
| **MAPK14** | **15.45** | **15.38** | **-0.07** | **15.49** | **15.05** | **-0.44** | **-0.36** |  | **MAPK14** | **15.59** | **15.43** | **-0.16** | **15.69** | **15.08** | **-0.62** | **-0.46** |
| **MCAM** | **25.22** | **25.34** | **0.13** | **25.27** | **25.24** | **-0.03** | **-0.16** |  | **MCAM** | **25.39** | **25.24** | **-0.15** | **25.38** | **25.29** | **-0.09** | **0.06** |
| **MHC2TA** | **16.74** | **16.72** | **-0.02** | **16.77** | **16.78** | **0.01** | **0.03** |  | **MHC2TA** | **16.71** | **16.63** | **-0.08** | **16.69** | **16.78** | **0.09** | **0.17** |
| **MIF** | **16.45** | **16.12** | **-0.32** | **16.33** | **16.08** | **-0.25** | **0.07** |  | **MIF** | **16.19** | **15.83** | **-0.35** | **16.21** | **15.83** | **-0.39** | **-0.03** |
| **MMP12** | **25.47** | **25.66** | **0.18** | **25.44** | **25.47** | **0.03** | **-0.15** |  | **MMP12** | **25.74** | **25.71** | **-0.02** | **25.70** | **25.76** | **0.06** | **0.08** |
| **MMP9** | **14.29** | **14.31** | **0.01** | **14.41** | **13.69** | **-0.72** | **-0.74** |  | **MMP9** | **14.68** | **14.48** | **-0.20** | **14.90** | **13.80** | **-1.10** | **-0.90** |
| **MNDA** | **12.60** | **12.72** | **0.12** | **12.67** | **12.64** | **-0.03** | **-0.15** |  | **MNDA** | **12.47** | **12.60** | **0.13** | **12.65** | **12.65** | **0.01** | **-0.12** |
| **MSH2** | **18.65** | **18.37** | **-0.28** | **18.47** | **18.24** | **-0.23** | **0.05** |  | **MSH2** | **18.52** | **18.17** | **-0.35** | **18.49** | **18.16** | **-0.33** | **0.02** |
| **MYC** | **18.71** | **18.56** | **-0.15** | **18.45** | **18.35** | **-0.10** | **0.05** |  | **MYC** | **18.64** | **18.36** | **-0.27** | **18.68** | **18.34** | **-0.34** | **-0.07** |
| **NAB2** | **20.63** | **20.69** | **0.06** | **20.57** | **20.68** | **0.11** | **0.06** |  | **NAB2** | **20.01** | **19.96** | **-0.05** | **19.97** | **20.12** | **0.16** | **0.21** |
| **NBEA** | **22.82** | **23.07** | **0.26** | **22.43** | **23.02** | **0.59** | **0.33** |  | **NBEA** | **22.55** | **22.67** | **0.12** | **22.49** | **22.75** | **0.26** | **0.14** |
| **NEDD4L** | **18.52** | **18.66** | **0.14** | **18.55** | **18.54** | **-0.01** | **-0.15** |  | **NEDD4L** | **18.89** | **18.78** | **-0.11** | **19.03** | **18.75** | **-0.29** | **-0.18** |
| **NEDD9** | **20.95** | **20.92** | **-0.04** | **21.19** | **20.95** | **-0.24** | **-0.20** |  | **NEDD9** | **20.79** | **20.70** | **-0.10** | **20.99** | **20.81** | **-0.17** | **-0.08** |
| **NFATC1** | **18.65** | **18.57** | **-0.08** | **19.19** | **19.16** | **-0.03** | **0.05** |  | **NFATC1** | **18.60** | **18.46** | **-0.14** | **18.44** | **18.40** | **-0.04** | **0.10** |
| **NFKB1** | **17.15** | **17.11** | **-0.04** | **17.08** | **16.96** | **-0.12** | **-0.09** |  | **NFKB1** | **17.24** | **17.09** | **-0.14** | **17.28** | **17.01** | **-0.27** | **-0.13** |
| **NME4** | **17.95** | **18.12** | **0.18** | **17.92** | **18.07** | **0.15** | **-0.03** |  | **NME4** | **17.83** | **17.92** | **0.08** | **17.80** | **17.81** | **0.01** | **-0.07** |
| **NRAS** | **17.43** | **17.20** | **-0.23** | **17.45** | **17.13** | **-0.32** | **-0.09** |  | **NRAS** | **17.40** | **17.05** | **-0.34** | **17.47** | **16.98** | **-0.49** | **-0.15** |
| **NUCKS1** | **17.77** | **17.58** | **-0.19** | **17.66** | **17.54** | **-0.12** | **0.08** |  | **NUCKS1** | **17.59** | **17.38** | **-0.20** | **17.61** | **17.46** | **-0.15** | **0.05** |
| **NUDT4** | **16.15** | **16.26** | **0.11** | **16.21** | **16.15** | **-0.06** | **-0.17** |  | **NUDT4** | **16.51** | **16.35** | **-0.16** | **16.70** | **16.36** | **-0.34** | **-0.18** |
| **PBX1** | **19.81** | **20.04** | **0.22** | **19.92** | **19.97** | **0.06** | **-0.17** |  | **PBX1** | **20.35** | **20.31** | **-0.04** | **20.59** | **20.35** | **-0.24** | **-0.20** |
| **PDE3B** | **17.50** | **17.54** | **0.04** | **17.35** | **17.49** | **0.13** | **0.09** |  | **PDE3B** | **17.31** | **17.35** | **0.03** | **17.29** | **17.44** | **0.15** | **0.11** |
| **PDGFA** | **20.29** | **20.55** | **0.25** | **20.44** | **20.44** | **0.00** | **-0.25** |  | **PDGFA** | **20.53** | **20.74** | **0.21** | **20.73** | **20.66** | **-0.07** | **-0.28** |
| **PLA2G7** | **19.22** | **19.49** | **0.27** | **19.28** | **19.67** | **0.39** | **0.13** |  | **PLA2G7** | **19.30** | **19.44** | **0.14** | **19.42** | **19.67** | **0.25** | **0.12** |
| **PLAUR** | **15.07** | **15.12** | **0.05** | **15.19** | **15.05** | **-0.15** | **-0.20** |  | **PLAUR** | **15.06** | **15.01** | **-0.05** | **15.19** | **15.23** | **0.04** | **0.09** |
| **PLEK2** | **18.39** | **18.62** | **0.23** | **18.52** | **18.40** | **-0.13** | **-0.36** |  | **PLEK2** | **18.87** | **18.87** | **0.00** | **19.18** | **18.91** | **-0.27** | **-0.27** |
| **PLXDC2** | **16.64** | **16.72** | **0.07** | **16.64** | **16.63** | **-0.01** | **-0.08** |  | **PLXDC2** | **16.64** | **16.60** | **-0.04** | **16.65** | **16.63** | **-0.02** | **0.02** |
| **PP2A** | **21.68** | **21.61** | **-0.07** | **21.53** | **21.58** | **0.05** | **0.12** |  | **PP2A** | **21.34** | **21.20** | **-0.14** | **21.40** | **21.42** | **0.02** | **0.17** |
| **PTEN** | **14.18** | **14.32** | **0.14** | **14.24** | **14.16** | **-0.09** | **-0.23** |  | **PTEN** | **14.27** | **14.36** | **0.09** | **14.40** | **14.37** | **-0.03** | **-0.12** |
| **PTGS2** | **17.40** | **17.57** | **0.16** | **17.49** | **17.76** | **0.27** | **0.11** |  | **PTGS2** | **17.61** | **17.80** | **0.19** | **17.65** | **18.17** | **0.52** | **0.33** |
| **PTPRC** | **12.47** | **12.39** | **-0.08** | **12.54** | **12.33** | **-0.21** | **-0.12** |  | **PTPRC** | **12.85** | **12.74** | **-0.12** | **12.92** | **12.68** | **-0.24** | **-0.12** |
| **PTPRK** | **22.63** | **22.76** | **0.13** | **22.64** | **22.69** | **0.04** | **-0.08** |  | **PTPRK** | **22.69** | **23.04** | **0.35** | **22.41** | **22.96** | **0.55** | **0.21** |
| **RBM5** | **16.41** | **16.42** | **0.02** | **16.34** | **16.28** | **-0.07** | **-0.08** |  | **RBM5** | **16.46** | **16.38** | **-0.08** | **16.53** | **16.44** | **-0.09** | **-0.01** |
| **RHOC** | **16.87** | **16.91** | **0.04** | **17.10** | **17.04** | **-0.06** | **-0.10** |  | **RHOC** | **16.58** | **16.61** | **0.03** | **16.71** | **16.69** | **-0.02** | **-0.05** |
| **RP51077B9.4** | **16.77** | **16.70** | **-0.07** | **16.81** | **16.49** | **-0.33** | **-0.26** |  | **RP51077B9.4** | **16.69** | **16.50** | **-0.19** | **16.78** | **16.38** | **-0.40** | **-0.20** |
| **S100A4** | **13.39** | **13.36** | **-0.03** | **13.40** | **13.22** | **-0.19** | **-0.16** |  | **S100A4** | **13.15** | **13.17** | **0.02** | **13.36** | **13.14** | **-0.22** | **-0.24** |
| **S100A6** | **15.14** | **15.17** | **0.04** | **15.13** | **14.99** | **-0.15** | **-0.18** |  | **S100A6** | **13.93** | **14.00** | **0.06** | **14.10** | **13.86** | **-0.24** | **-0.30** |
| **SCN3A** | **23.89** | **24.04** | **0.15** | **23.93** | **24.20** | **0.27** | **0.11** |  | **SCN3A** | **23.59** | **23.73** | **0.14** | **23.55** | **23.68** | **0.13** | **-0.01** |
| **SERPINA1** | **12.77** | **12.86** | **0.09** | **12.96** | **12.79** | **-0.17** | **-0.26** |  | **SERPINA1** | **12.82** | **12.86** | **0.05** | **12.97** | **12.82** | **-0.16** | **-0.21** |
| **SERPINE1** | **21.70** | **21.93** | **0.23** | **21.98** | **21.52** | **-0.45** | **-0.68** |  | **SERPINE1** | **21.89** | **21.91** | **0.03** | **22.05** | **21.80** | **-0.25** | **-0.28** |
| **SIAH2** | **13.89** | **13.96** | **0.08** | **14.05** | **13.87** | **-0.18** | **-0.25** |  | **SIAH2** | **14.42** | **14.26** | **-0.16** | **14.60** | **14.23** | **-0.38** | **-0.22** |
| **SLC4A1** | **14.00** | **14.23** | **0.23** | **14.10** | **14.27** | **0.17** | **-0.06** |  | **SLC4A1** | **14.72** | **14.68** | **-0.03** | **15.17** | **14.99** | **-0.19** | **-0.16** |
| **SOCS1** | **17.47** | **17.20** | **-0.27** | **17.48** | **17.11** | **-0.38** | **-0.11** |  | **SOCS1** | **17.49** | **17.20** | **-0.30** | **17.62** | **17.35** | **-0.27** | **0.03** |
| **SOCS3** | **18.31** | **18.25** | **-0.07** | **18.42** | **17.78** | **-0.65** | **-0.58** |  | **SOCS3** | **18.31** | **18.11** | **-0.20** | **18.46** | **17.65** | **-0.81** | **-0.61** |
| **SPARC** | **15.83** | **16.07** | **0.24** | **16.12** | **16.06** | **-0.06** | **-0.30** |  | **SPARC** | **16.05** | **16.13** | **0.09** | **16.27** | **16.02** | **-0.25** | **-0.33** |
| **ST14** | **18.15** | **18.14** | **-0.01** | **18.39** | **18.19** | **-0.20** | **-0.19** |  | **ST14** | **17.87** | **17.68** | **-0.18** | **17.98** | **17.76** | **-0.22** | **-0.03** |
| **TGFB1** | **13.10** | **13.05** | **-0.04** | **13.14** | **12.95** | **-0.19** | **-0.15** |  | **TGFB1** | **13.00** | **12.92** | **-0.09** | **13.11** | **12.89** | **-0.22** | **-0.13** |
| **THBS1** | **18.02** | **18.21** | **0.19** | **18.13** | **18.12** | **-0.01** | **-0.20** |  | **THBS1** | **17.89** | **17.94** | **0.05** | **17.99** | **17.97** | **-0.01** | **-0.06** |
| **TIMP1** | **14.57** | **14.64** | **0.07** | **14.82** | **14.64** | **-0.18** | **-0.25** |  | **TIMP1** | **14.94** | **14.96** | **0.02** | **15.07** | **14.81** | **-0.27** | **-0.29** |
| **TLK2** | **15.62** | **15.62** | **0.00** | **15.52** | **15.56** | **0.04** | **0.04** |  | **TLK2** | **15.68** | **15.63** | **-0.05** | **15.72** | **15.71** | **-0.01** | **0.04** |
| **TLR2** | **15.93** | **16.13** | **0.20** | **15.98** | **16.08** | **0.10** | **-0.09** |  | **TLR2** | **15.88** | **16.05** | **0.17** | **15.97** | **16.20** | **0.23** | **0.05** |
| **TLR4** | **15.00** | **15.12** | **0.12** | **15.13** | **14.85** | **-0.27** | **-0.40** |  | **TLR4** | **15.09** | **15.16** | **0.07** | **15.20** | **15.04** | **-0.16** | **-0.23** |
| **TLR9** | **18.14** | **18.15** | **0.01** | **18.11** | **18.14** | **0.04** | **0.03** |  | **TLR9** | **18.12** | **18.13** | **0.01** | **18.08** | **18.33** | **0.25** | **0.25** |
| **TMOD1** | **16.03** | **16.24** | **0.21** | **16.08** | **16.28** | **0.20** | **-0.01** |  | **TMOD1** | **16.65** | **16.61** | **-0.05** | **16.93** | **16.78** | **-0.15** | **-0.10** |
| **TNF** | **19.18** | **19.09** | **-0.09** | **19.14** | **19.02** | **-0.12** | **-0.03** |  | **TNF** | **19.34** | **19.17** | **-0.16** | **19.48** | **19.24** | **-0.24** | **-0.08** |
| **TNFRSF13B** | **21.85** | **21.40** | **-0.45** | **21.75** | **21.43** | **-0.32** | **0.13** |  | **TNFRSF13B** | **21.30** | **20.91** | **-0.39** | **21.34** | **20.72** | **-0.63** | **-0.23** |
| **TNFRSF1A** | **15.68** | **15.86** | **0.18** | **15.65** | **15.68** | **0.03** | **-0.16** |  | **TNFRSF1A** | **16.12** | **16.14** | **0.02** | **16.14** | **16.11** | **-0.03** | **-0.05** |
| **TNFRSF1B** | **13.40** | **13.43** | **0.03** | **13.40** | **13.41** | **0.01** | **-0.02** |  | **TNFRSF1B** | **13.26** | **13.28** | **0.01** | **13.38** | **13.47** | **0.09** | **0.07** |
| **TNFSF5** | **18.48** | **18.06** | **-0.42** | **18.23** | **17.99** | **-0.25** | **0.17** |  | **TNFSF5** | **18.61** | **18.15** | **-0.45** | **18.55** | **18.16** | **-0.39** | **0.07** |
| **TNFSF6** | **20.48** | **20.37** | **-0.12** | **20.55** | **20.56** | **0.01** | **0.12** |  | **TNFSF6** | **20.39** | **20.23** | **-0.15** | **20.53** | **20.03** | **-0.50** | **-0.35** |
| **TNS1** | **20.33** | **20.57** | **0.25** | **20.54** | **20.62** | **0.08** | **-0.17** |  | **TNS1** | **20.49** | **20.54** | **0.06** | **20.53** | **20.46** | **-0.07** | **-0.12** |
| **TOSO** | **16.69** | **16.36** | **-0.33** | **16.48** | **16.31** | **-0.16** | **0.17** |  | **TOSO** | **16.90** | **16.61** | **-0.30** | **16.90** | **16.73** | **-0.17** | **0.13** |
| **TP53** | **16.62** | **16.43** | **-0.19** | **16.59** | **16.40** | **-0.19** | **0.00** |  | **TP53** | **16.70** | **16.41** | **-0.29** | **16.78** | **16.45** | **-0.33** | **-0.04** |
| **TSPAN5** | **16.01** | **16.20** | **0.19** | **15.97** | **16.23** | **0.25** | **0.06** |  | **TSPAN5** | **16.48** | **16.47** | **-0.01** | **16.61** | **16.53** | **-0.08** | **-0.07** |
| **TXNRD1** | **16.97** | **16.97** | **-0.01** | **16.98** | **16.87** | **-0.11** | **-0.11** |  | **TXNRD1** | **16.95** | **16.90** | **-0.05** | **17.04** | **16.88** | **-0.16** | **-0.11** |
| **UBE2C** | **21.23** | **20.46** | **-0.77** | **21.31** | **20.17** | **-1.14** | **-0.37** |  | **UBE2C** | **21.62** | **20.55** | **-1.07** | **21.72** | **20.30** | **-1.43** | **-0.36** |
| **VEGF** | **23.34** | **23.31** | **-0.02** | **23.23** | **23.11** | **-0.12** | **-0.09** |  | **VEGF** | **23.64** | **23.43** | **-0.21** | **23.75** | **23.30** | **-0.46** | **-0.25** |
| **XK** | **17.89** | **18.15** | **0.26** | **17.99** | **18.18** | **0.19** | **-0.07** |  | **XK** | **18.68** | **18.57** | **-0.11** | **18.96** | **18.79** | **-0.17** | **-0.06** |
| **ZBTB10** | **23.26** | **23.45** | **0.19** | **23.20** | **23.18** | **-0.02** | **-0.20** |  | **ZBTB10** | **23.30** | **23.40** | **0.10** | **23.34** | **23.47** | **0.13** | **0.03** |

b.

|  |  |  |  |  |  |  |  |
| --- | --- | --- | --- | --- | --- | --- | --- |
| **Predictor Gene Name** | **Gene Expression Delta Ct Values Validation Dataset (N=210)** | | | | | | |
|  | **Grade 0-1 Diarrhea Pre Treatment N=154** | **Grade 0-1 Diarrhea Post Treatment N=154** | **Grade 0-1 Diarrhea Pre Post Difference** | **Grade 2-4 Diarrhea Pre Treatment N=56** | **Grade 2-4 Diarrhea Post Treatment N=56** | **Grade 2-4 Diarrhea Pre Post Difference** | **Grade 2-4 less Grade 0-1 Diarrhea Pre Post Difference** |
| **ADAM17** | **18.32** | **18.25** | **-0.07** | **18.38** | **18.31** | **-0.07** | **0.00** |
| **ALOX5** | **16.37** | **16.25** | **-0.12** | **16.54** | **16.06** | **-0.49** | **-0.37** |
| **ANLN** | **22.32** | **21.37** | **-0.96** | **22.38** | **21.22** | **-1.16** | **-0.21** |
| **APAF1** | **17.17** | **17.24** | **0.07** | **17.21** | **17.20** | **-0.01** | **-0.08** |
| **AXIN2** | **19.78** | **19.61** | **-0.17** | **19.79** | **19.69** | **-0.10** | **0.07** |
| **BAD** | **18.63** | **18.43** | **-0.20** | **18.66** | **18.43** | **-0.23** | **-0.04** |
| **BAX** | **15.77** | **15.67** | **-0.10** | **15.80** | **15.66** | **-0.15** | **-0.05** |
| **BLVRB** | **13.87** | **13.88** | **0.02** | **14.01** | **13.90** | **-0.11** | **-0.13** |
| **BPGM** | **17.21** | **17.13** | **-0.08** | **17.55** | **17.39** | **-0.16** | **-0.08** |
| **BRCA1** | **21.65** | **21.49** | **-0.15** | **21.66** | **21.47** | **-0.19** | **-0.04** |
| **C1QA** | **20.00** | **19.64** | **-0.36** | **20.30** | **19.31** | **-0.99** | **-0.62** |
| **CARD12** | **17.77** | **17.68** | **-0.09** | **17.95** | **17.34** | **-0.61** | **-0.53** |
| **CASP1** | **15.62** | **15.50** | **-0.13** | **15.76** | **15.50** | **-0.26** | **-0.13** |
| **CASP3** | **20.38** | **19.97** | **-0.41** | **20.51** | **20.03** | **-0.48** | **-0.06** |
| **CCL3** | **20.83** | **20.92** | **0.08** | **21.09** | **21.23** | **0.14** | **0.06** |
| **CCL5** | **13.19** | **12.96** | **-0.23** | **13.33** | **13.05** | **-0.28** | **-0.05** |
| **CCND1** | **23.21** | **23.43** | **0.22** | **23.40** | **23.62** | **0.22** | **0.00** |
| **CCR3** | **16.78** | **16.57** | **-0.21** | **16.99** | **17.41** | **0.42** | **0.63** |
| **CCR5** | **17.93** | **17.31** | **-0.62** | **18.08** | **17.47** | **-0.61** | **0.01** |
| **CCR7** | **15.70** | **15.54** | **-0.16** | **15.65** | **15.50** | **-0.15** | **0.01** |
| **CCR9** | **23.66** | **22.72** | **-0.94** | **23.75** | **22.70** | **-1.04** | **-0.10** |
| **CD19** | **19.56** | **19.65** | **0.10** | **19.61** | **19.59** | **-0.01** | **-0.11** |
| **CD28** | **17.65** | **16.83** | **-0.82** | **17.67** | **16.90** | **-0.77** | **0.04** |
| **CD4** | **15.36** | **15.16** | **-0.20** | **15.43** | **15.27** | **-0.15** | **0.04** |
| **CD40** | **19.92** | **19.86** | **-0.06** | **19.96** | **19.83** | **-0.14** | **-0.07** |
| **CD80** | **24.36** | **24.04** | **-0.32** | **24.27** | **24.13** | **-0.14** | **0.18** |
| **CD86** | **17.97** | **17.91** | **-0.06** | **18.07** | **17.88** | **-0.20** | **-0.14** |
| **CD8A** | **16.13** | **15.76** | **-0.37** | **16.17** | **15.94** | **-0.23** | **0.14** |
| **CD97** | **12.97** | **12.96** | **0.00** | **13.00** | **13.08** | **0.08** | **0.09** |
| **CDC25A** | **23.45** | **21.84** | **-1.61** | **23.64** | **21.45** | **-2.19** | **-0.58** |
| **CDH1** | **20.94** | **20.90** | **-0.04** | **21.10** | **20.92** | **-0.18** | **-0.14** |
| **CDK2** | **19.87** | **19.55** | **-0.32** | **19.93** | **19.51** | **-0.42** | **-0.10** |
| **CDKN1A** | **17.08** | **17.12** | **0.04** | **17.11** | **17.03** | **-0.09** | **-0.13** |
| **CDKN1B** | **15.11** | **15.00** | **-0.11** | **15.13** | **15.02** | **-0.11** | **0.00** |
| **CDKN2A** | **21.08** | **20.20** | **-0.88** | **21.18** | **20.13** | **-1.06** | **-0.18** |
| **CDKN2D** | **15.02** | **15.02** | **-0.01** | **15.06** | **14.92** | **-0.14** | **-0.13** |
| **CHPT1** | **16.81** | **16.71** | **-0.10** | **16.93** | **16.72** | **-0.21** | **-0.10** |
| **CNKSR2** | **22.10** | **21.88** | **-0.23** | **22.08** | **21.98** | **-0.10** | **0.13** |
| **CSF2** | **25.32** | **25.07** | **-0.25** | **25.33** | **25.09** | **-0.24** | **0.02** |
| **CTLA4** | **19.75** | **18.21** | **-1.55** | **19.68** | **18.21** | **-1.47** | **0.08** |
| **CTLA4SOL** | **25.37** | **25.29** | **-0.08** | **25.29** | **25.32** | **0.02** | **0.11** |
| **CTSD** | **13.10** | **12.98** | **-0.12** | **13.19** | **12.92** | **-0.26** | **-0.14** |
| **CXCL1** | **20.20** | **20.52** | **0.32** | **20.31** | **20.60** | **0.29** | **-0.03** |
| **CXCL10** | **24.24** | **23.85** | **-0.39** | **24.71** | **23.90** | **-0.81** | **-0.41** |
| **CXCR3** | **17.97** | **17.08** | **-0.89** | **18.06** | **17.20** | **-0.86** | **0.03** |
| **DLC1** | **23.92** | **23.91** | **-0.02** | **24.16** | **23.65** | **-0.51** | **-0.50** |
| **DPP4** | **18.91** | **18.80** | **-0.11** | **18.88** | **18.80** | **-0.08** | **0.03** |
| **E2F1** | **21.00** | **20.40** | **-0.60** | **21.28** | **20.21** | **-1.07** | **-0.47** |
| **EGR1** | **20.24** | **20.14** | **-0.10** | **20.24** | **20.04** | **-0.21** | **-0.11** |
| **ELA2** | **20.17** | **19.83** | **-0.34** | **20.07** | **19.40** | **-0.66** | **-0.32** |
| **ERBB2** | **23.13** | **22.91** | **-0.22** | **23.31** | **22.93** | **-0.38** | **-0.16** |
| **F5** | **18.08** | **17.80** | **-0.28** | **18.24** | **17.54** | **-0.70** | **-0.41** |
| **FAM210B** | **15.86** | **15.89** | **0.03** | **16.24** | **16.29** | **0.05** | **0.02** |
| **FCGR2B** | **12.38** | **12.45** | **0.07** | **12.51** | **12.43** | **-0.08** | **-0.15** |
| **FOS** | **15.94** | **16.20** | **0.26** | **15.99** | **16.32** | **0.33** | **0.07** |
| **FOXP3** | **20.53** | **19.62** | **-0.91** | **20.49** | **19.66** | **-0.83** | **0.08** |
| **FYN** | **15.25** | **15.04** | **-0.22** | **15.26** | **15.08** | **-0.19** | **0.03** |
| **GADD45A** | **19.97** | **19.66** | **-0.30** | **20.06** | **19.22** | **-0.84** | **-0.54** |
| **GLRX5** | **15.37** | **15.30** | **-0.07** | **15.60** | **15.31** | **-0.29** | **-0.22** |
| **GYPA** | **20.27** | **20.19** | **-0.08** | **20.58** | **20.48** | **-0.10** | **-0.02** |
| **GYPB** | **19.12** | **19.03** | **-0.08** | **19.52** | **19.40** | **-0.12** | **-0.04** |
| **GZMA** | **17.41** | **17.01** | **-0.39** | **17.50** | **17.14** | **-0.35** | **0.04** |
| **GZMB** | **17.25** | **17.12** | **-0.12** | **17.28** | **17.24** | **-0.03** | **0.09** |
| **HLADRA** | **12.59** | **12.50** | **-0.09** | **12.75** | **12.63** | **-0.13** | **-0.04** |
| **HMGA1** | **16.24** | **15.79** | **-0.44** | **16.23** | **15.74** | **-0.49** | **-0.05** |
| **HMGB1** | **18.17** | **17.89** | **-0.28** | **17.92** | **17.83** | **-0.09** | **0.19** |
| **HMOX1** | **16.26** | **16.36** | **0.10** | **16.42** | **16.34** | **-0.08** | **-0.19** |
| **HOXA10** | **23.02** | **22.97** | **-0.05** | **23.19** | **22.74** | **-0.45** | **-0.40** |
| **HSPA1A** | **14.95** | **14.86** | **-0.09** | **15.01** | **14.76** | **-0.25** | **-0.16** |
| **ICAM1** | **17.50** | **17.37** | **-0.13** | **17.66** | **17.41** | **-0.25** | **-0.12** |
| **ICOS** | **19.88** | **18.93** | **-0.95** | **19.79** | **18.96** | **-0.83** | **0.12** |
| **IFI16** | **14.78** | **14.54** | **-0.24** | **14.96** | **14.53** | **-0.43** | **-0.19** |
| **IFNG** | **23.34** | **22.78** | **-0.56** | **23.45** | **22.90** | **-0.55** | **0.01** |
| **IGF2BP2** | **16.68** | **16.64** | **-0.04** | **16.88** | **16.71** | **-0.17** | **-0.12** |
| **IGHG2** | **18.33** | **17.08** | **-1.25** | **18.65** | **16.77** | **-1.87** | **-0.62** |
| **IL10** | **23.37** | **22.90** | **-0.47** | **23.31** | **22.47** | **-0.84** | **-0.36** |
| **IL12B** | **25.72** | **25.70** | **-0.01** | **25.70** | **25.75** | **0.05** | **0.06** |
| **IL15** | **21.44** | **21.32** | **-0.12** | **21.64** | **21.30** | **-0.34** | **-0.22** |
| **IL18** | **21.79** | **21.81** | **0.02** | **21.84** | **21.73** | **-0.10** | **-0.13** |
| **IL18BP** | **17.42** | **17.27** | **-0.15** | **17.42** | **17.42** | **0.00** | **0.15** |
| **IL1B** | **16.57** | **16.59** | **0.02** | **16.73** | **16.64** | **-0.08** | **-0.11** |
| **IL1R1** | **20.71** | **20.82** | **0.11** | **20.74** | **20.57** | **-0.17** | **-0.28** |
| **IL1R2** | **16.23** | **16.41** | **0.17** | **16.25** | **16.15** | **-0.11** | **-0.28** |
| **IL1RN** | **16.69** | **16.73** | **0.04** | **16.91** | **16.63** | **-0.28** | **-0.32** |
| **IL2** | **25.43** | **25.39** | **-0.05** | **25.38** | **25.37** | **-0.01** | **0.04** |
| **IL23A** | **21.42** | **21.08** | **-0.35** | **21.47** | **21.19** | **-0.28** | **0.07** |
| **IL2RA** | **19.16** | **18.64** | **-0.51** | **19.16** | **18.79** | **-0.37** | **0.15** |
| **IL32** | **14.26** | **13.67** | **-0.58** | **14.27** | **13.74** | **-0.53** | **0.05** |
| **IL5** | **23.05** | **22.83** | **-0.22** | **23.40** | **23.27** | **-0.13** | **0.09** |
| **IL6** | **25.43** | **25.53** | **0.10** | **25.30** | **25.48** | **0.18** | **0.08** |
| **IL7R** | **15.52** | **15.30** | **-0.22** | **15.46** | **15.30** | **-0.16** | **0.06** |
| **IL8** | **22.12** | **22.80** | **0.67** | **22.23** | **23.49** | **1.26** | **0.58** |
| **INPP4B** | **17.84** | **17.51** | **-0.33** | **17.51** | **17.59** | **0.08** | **0.40** |
| **IRAK3** | **16.69** | **16.63** | **-0.07** | **16.80** | **16.35** | **-0.45** | **-0.39** |
| **IRF1** | **13.27** | **13.15** | **-0.12** | **13.38** | **13.23** | **-0.16** | **-0.04** |
| **ITGA4** | **14.56** | **14.19** | **-0.37** | **14.28** | **14.30** | **0.03** | **0.39** |
| **ITGAL** | **15.01** | **14.79** | **-0.22** | **15.06** | **14.88** | **-0.18** | **0.04** |
| **LARGE** | **22.87** | **23.03** | **0.16** | **22.80** | **22.89** | **0.09** | **-0.07** |
| **LCK** | **16.15** | **15.56** | **-0.59** | **16.12** | **15.62** | **-0.51** | **0.09** |
| **LGALS3** | **17.97** | **17.97** | **0.01** | **18.14** | **18.13** | **0.00** | **-0.01** |
| **LTA** | **20.08** | **19.93** | **-0.16** | **20.03** | **19.95** | **-0.08** | **0.08** |
| **MAPK14** | **15.59** | **15.43** | **-0.16** | **15.69** | **15.08** | **-0.62** | **-0.46** |
| **MCAM** | **25.39** | **25.24** | **-0.15** | **25.38** | **25.29** | **-0.09** | **0.06** |
| **MHC2TA** | **16.71** | **16.63** | **-0.08** | **16.69** | **16.78** | **0.09** | **0.17** |
| **MIF** | **16.19** | **15.83** | **-0.35** | **16.21** | **15.83** | **-0.39** | **-0.03** |
| **MMP12** | **25.74** | **25.71** | **-0.02** | **25.70** | **25.76** | **0.06** | **0.08** |
| **MMP9** | **14.68** | **14.48** | **-0.20** | **14.90** | **13.80** | **-1.10** | **-0.90** |
| **MNDA** | **12.47** | **12.60** | **0.13** | **12.65** | **12.65** | **0.01** | **-0.12** |
| **MSH2** | **18.52** | **18.17** | **-0.35** | **18.49** | **18.16** | **-0.33** | **0.02** |
| **MYC** | **18.64** | **18.36** | **-0.27** | **18.68** | **18.34** | **-0.34** | **-0.07** |
| **NAB2** | **20.01** | **19.96** | **-0.05** | **19.97** | **20.12** | **0.16** | **0.21** |
| **NBEA** | **22.55** | **22.67** | **0.12** | **22.49** | **22.75** | **0.26** | **0.14** |
| **NEDD4L** | **18.89** | **18.78** | **-0.11** | **19.03** | **18.75** | **-0.29** | **-0.18** |
| **NEDD9** | **20.79** | **20.70** | **-0.10** | **20.99** | **20.81** | **-0.17** | **-0.08** |
| **NFATC1** | **18.60** | **18.46** | **-0.14** | **18.44** | **18.40** | **-0.04** | **0.10** |
| **NFKB1** | **17.24** | **17.09** | **-0.14** | **17.28** | **17.01** | **-0.27** | **-0.13** |
| **NME4** | **17.83** | **17.92** | **0.08** | **17.80** | **17.81** | **0.01** | **-0.07** |
| **NRAS** | **17.40** | **17.05** | **-0.34** | **17.47** | **16.98** | **-0.49** | **-0.15** |
| **NUCKS1** | **17.59** | **17.38** | **-0.20** | **17.61** | **17.46** | **-0.15** | **0.05** |
| **NUDT4** | **16.51** | **16.35** | **-0.16** | **16.70** | **16.36** | **-0.34** | **-0.18** |
| **PBX1** | **20.35** | **20.31** | **-0.04** | **20.59** | **20.35** | **-0.24** | **-0.20** |
| **PDE3B** | **17.31** | **17.35** | **0.03** | **17.29** | **17.44** | **0.15** | **0.11** |
| **PDGFA** | **20.53** | **20.74** | **0.21** | **20.73** | **20.66** | **-0.07** | **-0.28** |
| **PLA2G7** | **19.30** | **19.44** | **0.14** | **19.42** | **19.67** | **0.25** | **0.12** |
| **PLAUR** | **15.06** | **15.01** | **-0.05** | **15.19** | **15.23** | **0.04** | **0.09** |
| **PLEK2** | **18.87** | **18.87** | **0.00** | **19.18** | **18.91** | **-0.27** | **-0.27** |
| **PLXDC2** | **16.64** | **16.60** | **-0.04** | **16.65** | **16.63** | **-0.02** | **0.02** |
| **PP2A** | **21.34** | **21.20** | **-0.14** | **21.40** | **21.42** | **0.02** | **0.17** |
| **PTEN** | **14.27** | **14.36** | **0.09** | **14.40** | **14.37** | **-0.03** | **-0.12** |
| **PTGS2** | **17.61** | **17.80** | **0.19** | **17.65** | **18.17** | **0.52** | **0.33** |
| **PTPRC** | **12.85** | **12.74** | **-0.12** | **12.92** | **12.68** | **-0.24** | **-0.12** |
| **PTPRK** | **22.69** | **23.04** | **0.35** | **22.41** | **22.96** | **0.55** | **0.21** |
| **RBM5** | **16.46** | **16.38** | **-0.08** | **16.53** | **16.44** | **-0.09** | **-0.01** |
| **RHOC** | **16.58** | **16.61** | **0.03** | **16.71** | **16.69** | **-0.02** | **-0.05** |
| **RP51077B9.4** | **16.69** | **16.50** | **-0.19** | **16.78** | **16.38** | **-0.40** | **-0.20** |
| **S100A4** | **13.15** | **13.17** | **0.02** | **13.36** | **13.14** | **-0.22** | **-0.24** |
| **S100A6** | **13.93** | **14.00** | **0.06** | **14.10** | **13.86** | **-0.24** | **-0.30** |
| **SCN3A** | **23.59** | **23.73** | **0.14** | **23.55** | **23.68** | **0.13** | **-0.01** |
| **SERPINA1** | **12.82** | **12.86** | **0.05** | **12.97** | **12.82** | **-0.16** | **-0.21** |
| **SERPINE1** | **21.89** | **21.91** | **0.03** | **22.05** | **21.80** | **-0.25** | **-0.28** |
| **SIAH2** | **14.42** | **14.26** | **-0.16** | **14.60** | **14.23** | **-0.38** | **-0.22** |
| **SLC4A1** | **14.72** | **14.68** | **-0.03** | **15.17** | **14.99** | **-0.19** | **-0.16** |
| **SOCS1** | **17.49** | **17.20** | **-0.30** | **17.62** | **17.35** | **-0.27** | **0.03** |
| **SOCS3** | **18.31** | **18.11** | **-0.20** | **18.46** | **17.65** | **-0.81** | **-0.61** |
| **SPARC** | **16.05** | **16.13** | **0.09** | **16.27** | **16.02** | **-0.25** | **-0.33** |
| **ST14** | **17.87** | **17.68** | **-0.18** | **17.98** | **17.76** | **-0.22** | **-0.03** |
| **TGFB1** | **13.00** | **12.92** | **-0.09** | **13.11** | **12.89** | **-0.22** | **-0.13** |
| **THBS1** | **17.89** | **17.94** | **0.05** | **17.99** | **17.97** | **-0.01** | **-0.06** |
| **TIMP1** | **14.94** | **14.96** | **0.02** | **15.07** | **14.81** | **-0.27** | **-0.29** |
| **TLK2** | **15.68** | **15.63** | **-0.05** | **15.72** | **15.71** | **-0.01** | **0.04** |
| **TLR2** | **15.88** | **16.05** | **0.17** | **15.97** | **16.20** | **0.23** | **0.05** |
| **TLR4** | **15.09** | **15.16** | **0.07** | **15.20** | **15.04** | **-0.16** | **-0.23** |
| **TLR9** | **18.12** | **18.13** | **0.01** | **18.08** | **18.33** | **0.25** | **0.25** |
| **TMOD1** | **16.65** | **16.61** | **-0.05** | **16.93** | **16.78** | **-0.15** | **-0.10** |
| **TNF** | **19.34** | **19.17** | **-0.16** | **19.48** | **19.24** | **-0.24** | **-0.08** |
| **TNFRSF13B** | **21.30** | **20.91** | **-0.39** | **21.34** | **20.72** | **-0.63** | **-0.23** |
| **TNFRSF1A** | **16.12** | **16.14** | **0.02** | **16.14** | **16.11** | **-0.03** | **-0.05** |
| **TNFRSF1B** | **13.26** | **13.28** | **0.01** | **13.38** | **13.47** | **0.09** | **0.07** |
| **TNFSF5** | **18.61** | **18.15** | **-0.45** | **18.55** | **18.16** | **-0.39** | **0.07** |
| **TNFSF6** | **20.39** | **20.23** | **-0.15** | **20.53** | **20.03** | **-0.50** | **-0.35** |
| **TNS1** | **20.49** | **20.54** | **0.06** | **20.53** | **20.46** | **-0.07** | **-0.12** |
| **TOSO** | **16.90** | **16.61** | **-0.30** | **16.90** | **16.73** | **-0.17** | **0.13** |
| **TP53** | **16.70** | **16.41** | **-0.29** | **16.78** | **16.45** | **-0.33** | **-0.04** |
| **TSPAN5** | **16.48** | **16.47** | **-0.01** | **16.61** | **16.53** | **-0.08** | **-0.07** |
| **TXNRD1** | **16.95** | **16.90** | **-0.05** | **17.04** | **16.88** | **-0.16** | **-0.11** |
| **UBE2C** | **21.62** | **20.55** | **-1.07** | **21.72** | **20.30** | **-1.43** | **-0.36** |
| **VEGF** | **23.64** | **23.43** | **-0.21** | **23.75** | **23.30** | **-0.46** | **-0.25** |
| **XK** | **18.68** | **18.57** | **-0.11** | **18.96** | **18.79** | **-0.17** | **-0.06** |
| **ZBTB10** | **23.30** | **23.40** | **0.10** | **23.34** | **23.47** | **0.13** | **0.03** |
|  |  |  |  |  |  |  |  |
|  |  |  |  |  |  |  |  |
|  |  |  |  |  |  |  |  |
|  |  |  |  |  |  |  |  |
|  |  |  |  |  |  |  |  |
|  |  |  |  |  |  |  |  |
|  |  |  |  |  |  |  |  |
|  |  |  |  |  |  |  |  |
|  |  |  |  |  |  |  |  |
|  |  |  |  |  |  |  |  |
|  |  |  |  |  |  |  |  |
|  |  |  |  |  |  |  |  |
|  |  |  |  |  |  |  |  |
|  |  |  |  |  |  |  |  |
|  |  |  |  |  |  |  |  |
|  |  |  |  |  |  |  |  |
|  |  |  |  |  |  |  |  |
|  |  |  |  |  |  |  |  |
|  |  |  |  |  |  |  |  |
|  |  |  |  |  |  |  |  |
|  |  |  |  |  |  |  |  |
|  |  |  |  |  |  |  |  |
|  |  |  |  |  |  |  |  |
|  |  |  |  |  |  |  |  |
|  |  |  |  |  |  |  |  |
|  |  |  |  |  |  |  |  |
|  |  |  |  |  |  |  |  |
|  |  |  |  |  |  |  |  |
|  |  |  |  |  |  |  |  |
|  |  |  |  |  |  |  |  |
|  |  |  |  |  |  |  |  |
|  |  |  |  |  |  |  |  |
|  |  |  |  |  |  |  |  |
|  |  |  |  |  |  |  |  |
|  |  |  |  |  |  |  |  |
